# Programming peptide-oligonucleotide nano-assembly for engineering of neoantigen vaccine with potent immunogenicity

**DOI:** 10.7150/thno.93395

**Published:** 2024-03-17

**Authors:** Zhichu Xiang, Jianhua Lu, Shangrui Rao, Chenxing Fu, Yuying Yao, Yongdong Yi, Yang Ming, Weijian Sun, Weisheng Guo, Xiaoyuan Chen

**Affiliations:** 1 Department of Gastrointestinal Surgery, The Second Affiliated Hospital and Yuying Children's Hospital of Wenzhou Medical University, Wenzhou 325027, China.; 2 Departments of Diagnostic Radiology, Surgery, Chemical and Biomolecular Engineering, and Biomedical Engineering, Yong Loo Lin School of Medicine and College of Design and Engineering, National University of Singapore, Singapore 119074, Singapore.; 3 Clinical Imaging Research Centre, Centre for Translational Medicine, Yong Loo Lin School of Medicine, National University of Singapore, Singapore 117599, Singapore.; 4 Nanomedicine Translational Research Program, Yong Loo Lin School of Medicine, National University of Singapore, Singapore 117597, Singapore.; 5 Institute of Molecular and Cell Biology, Agency for Science, Technology, and Research (A*STAR), 61 Biopolis Drive, Proteos, Singapore 138673, Singapore.; 6 Department of Minimally Invasive Interventional Radiology, the State Key Laboratory of Respiratory Disease, School of Biomedical Engineering & The Second Affiliated Hospital, Guangzhou Medical University, Guangzhou, 510260, China.

**Keywords:** neoantigen, assembly, nanovaccine, peptide programming, immunotherapy

## Abstract

**Background:** Neoantigen nanovaccine has been recognized as a promising treatment modality for personalized cancer immunotherapy. However, most current nanovaccines are carrier-dependent and the manufacturing process is complicated, resulting in potential safety concerns and suboptimal codelivery of neoantigens and adjuvants to antigen-presenting cells (APCs).

**Methods:** Here we report a facile and general methodology for nanoassembly of peptide and oligonucleotide by programming neoantigen peptide with a short cationic module at N-terminus to prepare nanovaccine. The programmed peptide can co-assemble with CpG oligonucleotide (TLR9 agonist) into monodispersed nanostructures without the introduction of artificial carrier.

**Results:** We demonstrate that the engineered nanovaccine promoted the codelivery of neoantigen peptides and adjuvants to lymph node-residing APCs and instigated potent neoantigen-specific T-cell responses, eliciting neoantigen-specific antitumor immune responses with negligible systemic toxicity. Furthermore, the antitumor T-cell immunity is profoundly potentiated when combined with anti-PD-1 therapy, leading to significant inhibition or even complete regression of established melanoma and MC-38 colon tumors.

**Conclusions:** Collectively, this work demonstrates the feasibility and effectiveness of personalized cancer nanovaccine preparation with high immunogenicity and good biosafety by programming neoantigen peptide for nanoassembly with oligonucleotides without the aid of artificial carrier.

## Introduction

Recent years have witnessed encouraging clinical breakthroughs in tumor immunotherapy, the treatment strategy that harnesses the patient's own immune system to fight against cancer 1-8. While chimeric antigen receptor T (CAR-T) cells and immune checkpoint inhibitors (ICIs) have triggered tumor remission in a subset of patients, the therapeutic efficacy is limited and some of them show adverse reactions 9-12. This may be attributed to the deficiency of employing tumor-overexpressed or re-expressed antigens as targets that are not exclusive to tumor cells, but also shared by normal tissues at lower levels 13,14. The non-specificity of these conventional vaccines against healthy tissues can lead to suboptimal selectivity to tumor tissues, resulting in off-tumor side effects and autoimmune toxicities 15,16. Therefore, developing antitumor nanovaccines that are not only clinically effective but also show negligible side effects to normal tissue is highly desired.

Neoantigens refer to the antigens derived from non-synonymous somatic mutations that exclusively occur in tumor cells and identified through whole-exome sequencing of tumor genome 17-19. The high immunogenicity and tumor exclusiveness of neoantigens make them excellent targets for designing personalized therapeutic nanovaccine. Currently, the nanovaccine based on codelivery of neoantigen and adjuvants have achieved encouraging outcomes in both animal models and some patients 20-26, which is demonstrated by the potent antitumor immune response of their clinical investigations 27-30. Despite the impressive progress, most of the current nanovaccines are developed based on either codelivery of neoantigen peptides (Ag) and adjuvants using polymer scaffolds or nanocarriers 31-34, causing some potential safety concerns and inefficiency in co-delivery of neoantigen peptides and adjuvants to APCs. Moreover, some nanocarriers are complicatedly designed and require complex manufacturing processes, which makes the quality control and scale-up manufacturing very challenging. Therefore, designing nanovaccine that integrates both neoantigen peptides and immune adjuvants in a simple procedure without introducing artificial carrier is highly desirable for improving the outcomes of neoantigen-specific tumor immunotherapy.

**Figure 1:**
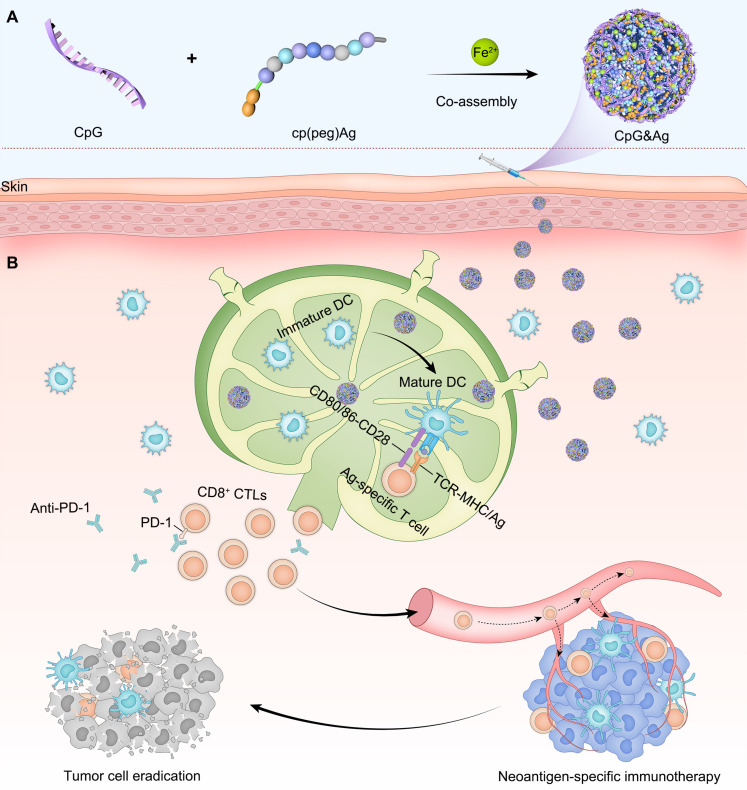
Design of neoantigen nanovaccine for personalized tumor immunotherapy. **(A)** The CpG&Ag is engineered by co-assembly of programmed neoantigen peptide and CpG agonist through a one-pot reaction process. **(B)** Following subcutaneous injection, CpG&Ag is efficiently accumulated in draining lymph nodes (dLNs), enabling codelivery of neoantigen and agonist to APCs, leading to DC maturation and elicitation of neoantigen-specific immune responses. The tumor progression is markedly restrained and even completely regressed when combined with ICB therapy.

The nano-assembly of CpG oligonucleotide, a potent TLR9 agonist 35 and neoantigen peptide, without carrier for codelivery is challenging due to their significant differences in both sequences and structures 36. Recent progress in coordination-driven self-assembly has provided a feasible strategy for delivering small molecules and nucleic acid therapeutics 37-41. Herein we report a facile strategy to construct the neoantigen nanovaccine for antitumor immunotherapy based on metal ions-coordinated co-assembly of neoantigen peptide and TLR9 agonist. Specifically, neoantigen peptide is programmed for the nano-assembly by grafting a phosphorylated serine-functionalized short cationic module (cp) at N-terminus through a poly(ethylene glycol) (PEG5) linker, which not only improved the solubility of neoantigen peptide but also increased the interaction between neoantigen and CpG agonist. Additionally, the phosphorylated serine (^P^S) incorporated at N-terminus increased the coordination interaction between metal ions and peptide 42. We demonstrated that both short and long neoantigen peptides (antigen peptide from ovalbumin: OVA_257-264_, and neoantigen peptide in MC-38 cell: Adgpk with the mutation (ASMTN**R**ELM → ASMTN**M**ELM)) could co-assemble with CpG agonist into monodispersed nanoparticles (termed as CpG&Ag) by employing the programming strategy (**Figure 1A**) 18. The engineered nanovaccine promoted the codelivery of neoantigen and agonist to APCs and elicit potent neoantigen-specific antitumor immune responses with negligible systemic toxicity, leading to the eradication of some tumors when combined with immune checkpoint blockade (ICB) therapy (**Figure 1B**). Therefore, our strategy offers a simple and promising technologic platform for personalized cancer nanovaccine development.

## Results and Discussion

### Preparation and characterization of CpG&Ag

**Figure 2:**
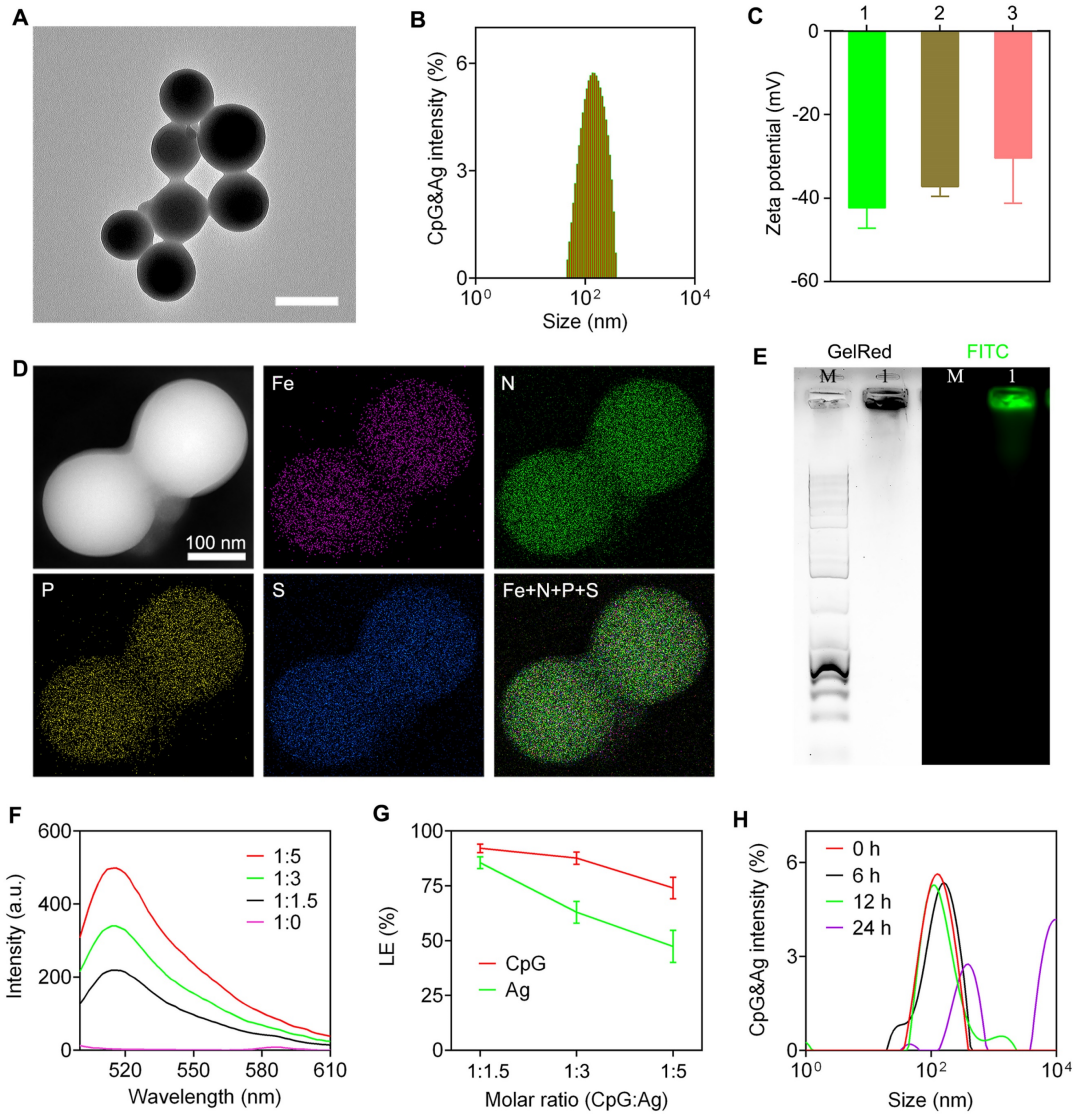
Characterization of nanovaccine CpG&Ag. **(A)** TEM image, and **(B)** size distribution of CpG&cp(peg)OVA. Scale bar, 200 nm. **(C)** Zeta potentials of 1: CpG-Fe, 2: CpG&cp(peg)OVA, 3: CpG&cp(peg)Adpgk. **(D)** HAADF-STEM and elemental mapping images of CpG&cp(peg)OVA. **(E)** Agarose gel electrophoresis images of CpG&cp(peg)OVA (FITC-labeled, lane M: 1kb DNA ladder, lane 1: CpG&cp(peg)OVA, left: GelRed imaging, right: FITC fluorescence imaging). **(F)** Fluorescence spectra of CpG&cp(peg)OVA prepared with varying CpG:cp(peg)OVA (FITC-labeled) ratios. **(G)** Loading efficiency of CpG and Ag (Ag: cp(peg)OVA) at varying CpG:Ag ratios. **(H)** Hydrodynamic size distribution of CpG&Ag (Ag: cp(peg)OVA) after incubated in HEPES buffer (pH 7.4) for different times. Data are presented as means ± s.d. (n = 3).

The nanovaccine CpG&Ag was engineered *via* a one-pot nano-assembly reaction of CpG, Ag (cp(peg)OVA or cp(peg)Adpgk) in the presence of Fe^2+^ (CpG:Ag ratio 1:1.5) at 95 ℃ for 2 h. Transmission electron microscopy (TEM) images showed the spherical and monodispersed nanostructures of both CpG&cp(peg)OVA and CpG&cp(peg)Adpgk (**Figure 2A** and **Figure S1A**). Dynamic light scattering (DLS) results showed that the average sizes of the CpG&cp(peg)OVA and CpG&cp(peg)Adpgk were 153.6 ± 50.3 nm (PDI: 0.21) and 165.8 ± 46.4 nm (PDI: 0.27), respectively, which were slightly smaller than CpG-Fe (181.7 ± 37.6 nm, PDI: 0.18) prepared in the absence of neoantigen peptide (**Figure 2B** and **Figure S1B**). Zeta potential analysis revealed that the neoantigen peptide and CpG co-assembled nanovaccines (CpG&cp(peg)OVA: -37.2 mV; CpG&cp(peg)Adpgk: -30.4 mV) had higher surface charges than CpG-Fe (-42.3 mV), which could be attributed to the positive charge of cp-grafted neoantigen peptide (**Figure 2C**). Elemental mapping analysis using high-angle annular dark-field scanning transmission electron microscopy (HAADF-STEM) demonstrated that Fe, phosphorus (from CpG and phosphorylated serine) and sulfur (from cysteine in Ag) were homogeneously distributed throughout the nanoparticles in contrast to no sulfur signal in CpG-Fe (**Figure 2D** and **Figure S2**), indicating successful co-assembly of neoantigen peptide and agonist into nanovaccine. To further confirm that, we prepared the nanovaccine with FITC-labeled Ag peptide (^P^SKRKKK(peg5)CSIINFEK_(FITC)_L). Agarose gel electrophoresis results showed complete retardation of CpG and the GelRed signal (from CpG) colocalized well with FITC signal (from Ag) for the prepared nanoparticles (**Figure 2E**). Most importantly, the preparation procedure had negligible effect on the peptide sequence, which is confirmed by ESI-MS (**Figure S3**). To evaluate the assembly and loading efficiency of Ag (^P^SKRKKK(peg5)CSIINFEK_(FITC)_L) and CpG, different ratios of CpG:Ag were supplemented for the nanoparticle preparation. Notably, the nano-assemblies prepared in higher ratios of Ag:CpG contained some small agglomerates or even some hybrid nanofibers in addition to spherical nanoparticles (**Figure S4**), which could be attributed to the interference of excess neoantigen peptide to the coordination interactions between Fe^2+^ and CpG. Fluorescence spectra of the nano-assemblies prepared at different ratios of CpG:Ag revealed the gradual increase of FITC signal when more Ag was added (**Figure 2F**). Further analysis showed that the loading efficiency of both CpG and Ag decreased respectively from 92.1% and 85.5% to 74.0% and 47.4% upon decreasing the CpG:Ag ratio from 1:1.5 to 1:5. We next investigated the stability of the prepared nanovaccine in physiological conditions. As shown in **figure S5** and **figure 2H**, the CpG&Ag maintained their spherical structure even after incubation in HEPES buffer for 6 h, demonstrating good biostability of the nanovaccine. Notably, negligible cytotoxicity of CpG&Ag to both DC2.4 cells and macrophages was observed after 24 h treatment (**Figure S6**), indicating good biosafety of our nanovaccine.

### CpG&Ag-mediated durable Ag presentation and immunostimulation

**Figure 3:**
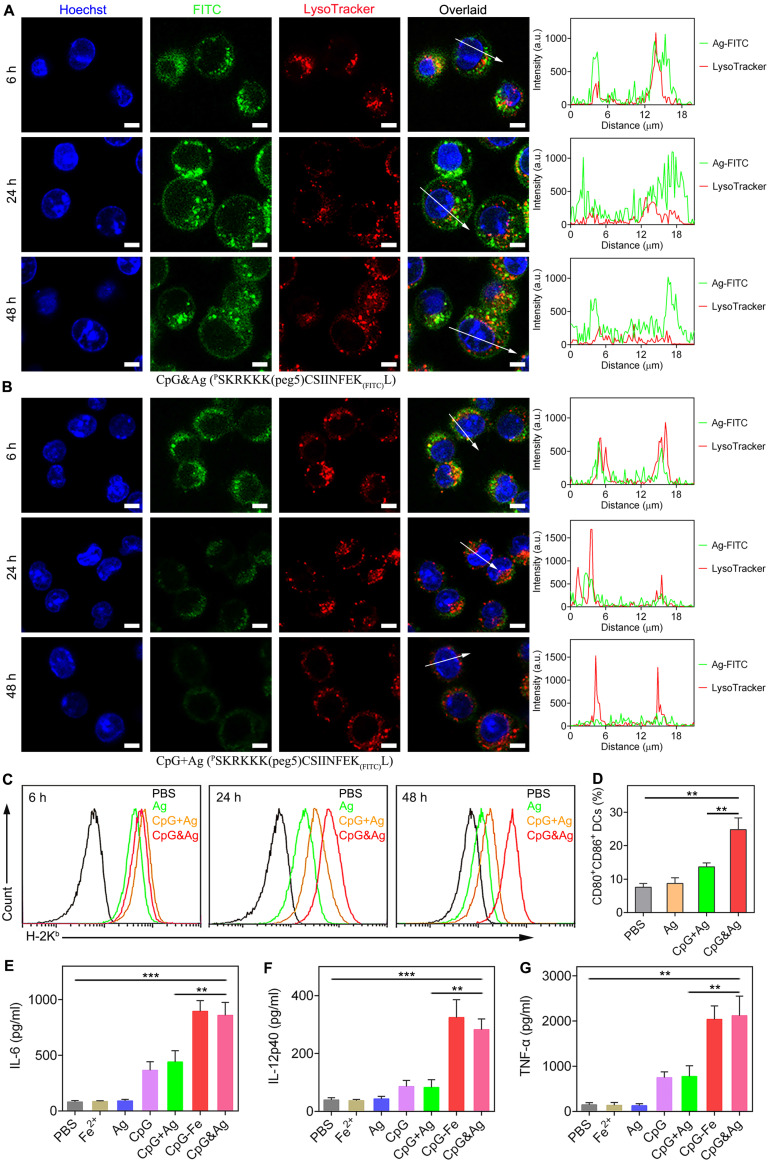
Nanovaccine-mediated durable antigen presentation and immunostimulation. Confocal fluorescence images of DC2.4 cells after treatment with **(A)** CpG&Ag and **(B)** CpG + Ag (Ag: ^P^SKRKKK(peg5)CSIINFEK_(FITC)_L) for 6, 24 and 48 h (CpG: 250 nM, Ag: 380 nM). Fluorescence intensity of FITC-labeled Ag peptide and lysosome (red) signals across the regions indicated with white arrows were analyzed and demonstrated in line plots. **(C)** Flow cytometry evaluation of SIINFEKL/H-2K^b^ complex on DCs after treatment with Ag, CpG + Ag, or CpG&Ag (Ag: ^P^SKRKKK(peg5)CSIINFEK_(FITC)_L) for 4, 24 and 48 h, respectively (CpG: 250 nM, Ag: 380 nM). **(D)** The expression of CD80 and CD86 on DC2.4 cells after the indicated treatment (CpG: 250 nM). The concentrations of **(E)** IL-6, **(F)** IL-12p40, and **(G)** TNF-α in culture medium secreted from DC2.4 cells after indicated treatments. Scale bars, 5 μm. Data are presented as means ± s.d. (n = 3). **P < 0.01, ***P < 0.001, (Student's t test).

After confirming the successful engineering of CpG&Ag, we investigated the cellular uptake and localization of Ag and CpG delivered by the nanovaccine. DC2.4 cells and RAW264.7 macrophages were incubated with CpG&cp(peg)OVA (FITC-labeled) or free CpG + cp(peg)OVA (FITC-labeled) mixture. Confocal fluorescence imaging showed that the nanovaccine-treated DC2.4 cells displayed robust FITC signal that colocalized well with endo/lysosomes at 6 h post-treatment. Interestingly, strong cp(peg)OVA (FITC-labeled) signal was detected on cell membranes at 24 h (**Figure 3A**, **Figure S7 and S8**) and maintained up to 48 h post-treatment (**Figure 3A** and **Figure S7**). In contrast, only weak cp(peg)OVA (FITC-labeled) signal was observed on cell membranes for the CpG + cp(peg)OVA (FITC-labeled)-treated cells at 6 h, and the signal gradually dimmed after 24 or 48 h of incubation (**Figure 3B**, **Figure S7** and **S8B**).

Encouraged by the efficient intracellular codelivery of adjuvants and antigens, we sought to evaluate the OVA presentation on DC cell membrane induced by CpG&cp(peg)OVA (FITC-labeled). The cells were stained with an antibody that specifically recognizes SIINFEKL/H-2K^b^ complexes after indicated treatment. Flow cytometry analysis revealed that the incubation with CpG&cp(peg)OVA (FITC-labeled) for 6 h led to efficient presentation of SIINFEKL on DCs (**Figure 3C**). Notably, the presentation increased and maintained up to 48 h, and showed much higher levels than the control groups, demonstrating efficient and sustained antigen presentation induced by the nanovaccine. In contrast, the cells treated with free Ag or CpG + Ag mixture demonstrated high level of SIINFEKL presentation at 6 h. However, the presentation gradually decreased in the following 24 h and showed precipitous reduction at 48 h, which could be attributed to the rapid degradation or disassociation of antigen from MHC molecules. To further evaluate the immunostimulation of CpG&Ag on APCs, DC2.4 cells were treated with indicated vaccine formulations. Flow cytometry analysis showed that the treatment with our nanovaccine led to up-regulation of costimulatory factors CD80 and CD86 compared with control groups (**Figure 3D** and **Figure S9**). ELISA results showed that much higher levels of IL-6, interleukin-12 (IL-12), and tumor necrosis factor-α (TNF-α) were secreted treatment with our nanovaccine in comparison to free Ag or CpG + Ag treatment (**Figure 3E-G**), indicating DC maturation was potently stimulated by CpG&Ag. Collectively, the nanovaccine promoted intracellular codelivery of both CpG and antigen and further mediated their durable release in endo/lysosomes, contributing to the sustained antigen presentation, potent immunostimulation, and potentially enhanced CD8^+^ T cell cross-priming.

### Lymphatic and intracellular co-delivery of CpG and Ag to APCs *in vivo*

**Figure 4:**
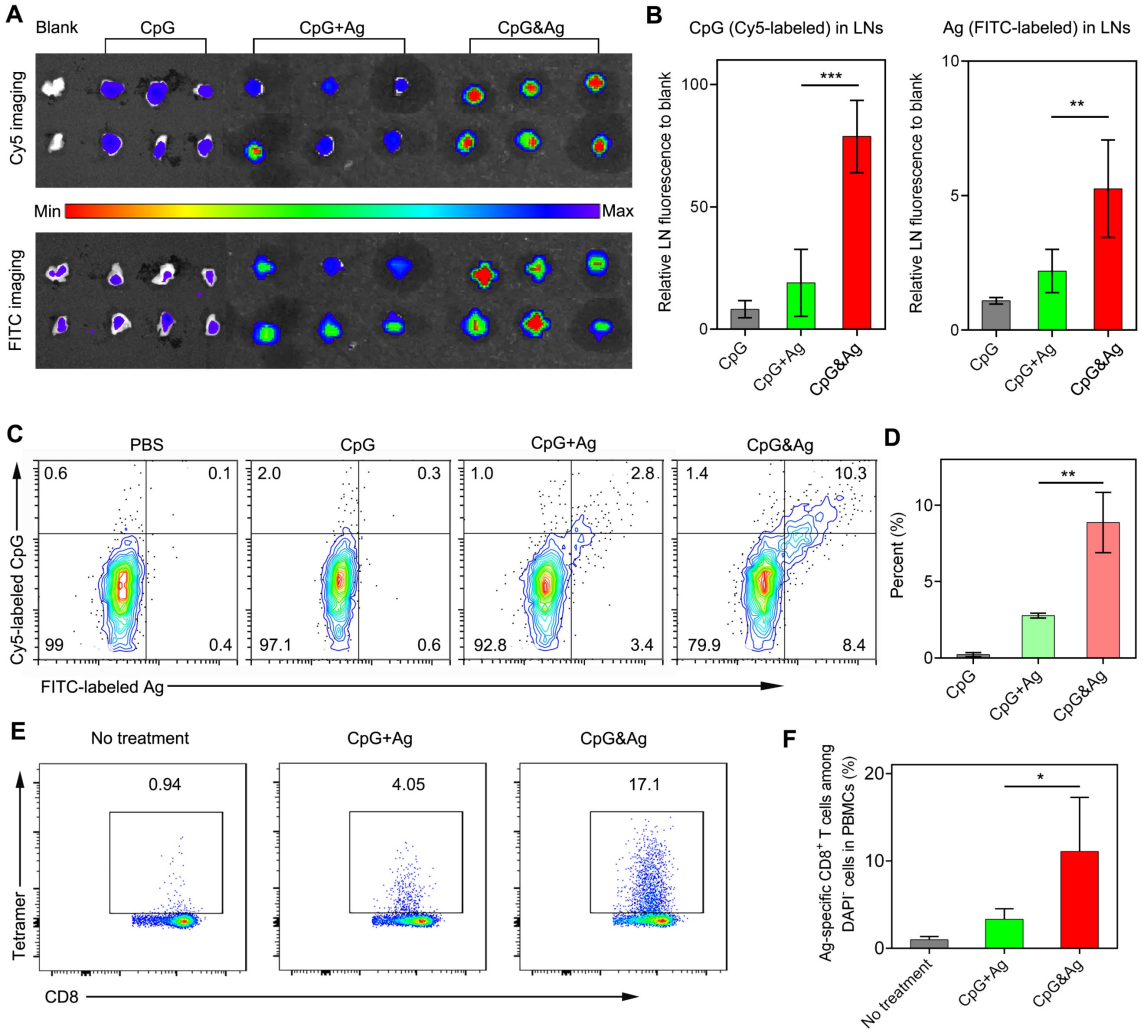
Nanovaccine-mediated co-delivery of CpG and Ag to dLN-residing APCs with high efficiency. **(A)** Fluorescence imaging and **(B)** signal quantification of Cy5 and FITC intensity of inguinal dLNs at 12 h after subcutaneously injected with CpG, CpG + Ag or CpG&Ag (CpG: Cy5-labeled, 1 nmol per mouse; Ag: cp(peg)CSIINFEK_(FITC)_L, 3.5 μg per mouse) at the tail base, respectively. **(C)** Flow cytometry analysis and **(D)** quantification of Cy5^+^/FITC^+^ dLN-residing APCs demonstrating the efficient codelivery of CpG (Cy5-labeled) and Ag (FITC-labeled) to inguinal dLN-residing DCs at 12 h after the nanovaccine treatments. **(E)** Flow cytometry scatter plots and **(F)** the frequency of Ag-specific CD8^+^ T cells in peripheral blood collected from the mice 7 days after the third vaccination with indicated vaccine formulations (CpG: 3 nmol, Ag: ^P^SKRKKK(peg5)CSIINFEKL, 10 μg). Data are presented as means ± s.d. (n = 3). *P < 0.05, **P < 0.01, ***P < 0.001.

Having examined the durable antigen presentation and robust immunostimulation of CpG&Ag *in vitro*, we sought to investigate the draining lymph node (dLN) accumulation and co-delivery of CpG and Ag to dLN-residing APCs mediated by the nanovaccine *in vivo*. Two groups of C57BL/6 mice were subcutaneously administered with CpG&Ag and free CpG + Ag mixture (CpG: Cy5-labeled, Ag: ^P^SKRKKK(peg5)CSIINFEK_(FITC)_L) at the tail base, respectively. The inguinal dLNs were collected for fluorescence imaging at 12 h post-injection. As shown in **Figure 4a**, strong FITC and Cy5 signals could be detected in the inguinal dLNs collected from CpG&Ag group in contrast to the marginal FITC and Cy5 fluorescence increase in CpG + Ag group. Quantification analysis revealed that the nanovaccine treatment produced about 4.4- and 1.8-fold higher Cy5 and FITC fluorescence in inguinal dLNs than CpG + Ag treatment, respectively (**Figure 4A-B**), suggesting more efficient dLN accumulation of CpG&Ag than the free vaccine formulation. To further evaluate the co-delivery of CpG and Ag to APCs, which is desirable for the robust immunostimulation, the fluorescence signals in dLN-residing DCs were examined. Flow cytometry analysis showed that much higher proportion (9.7%) of CpG^+^Ag^+^ DCs were produced after CpG&Ag-treatment, which is about 3.7-fold higher than that in CpG + Ag group (**Figure 4C-D**), confirming the nanovaccine-mediated efficient co-delivery of antigen peptide and agonist to dLN-residing APCs *in vivo*. Notably, no splenomegaly was observed in comparison to control groups after the vaccination (**Figure S10**).

After verifying the efficient codelivery of CpG and Ag to dLNs mediated by the nanovaccine, the presentation of SIINFEKL on CD8^+^ T cells in peripheral blood was evaluated after the mice were vaccinated with CpG&Ag or CpG + Ag (**Figure S11**). As shown in **Figure 4E**-**F**, the nanovaccine formulation elicited ~17% of SIINFEKL-specific CD8^+^ T cells after the treatment, demonstrating robust T-cell cross-priming induced by CpG&Ag. In contrast, the immunization with the mixture of free Ag peptide and CpG induced only ~4% Ag-specific CD8^+^ T cells. Additionally, the elevated expressions of CD80 and CD86 were detected in LN DCs and macrophages after immunized with the nanovaccine, demonstrating increased APC activation *in vivo* (**Figure S12**).

### Tumor immunosuppression of CpG&Ag combined with ICB

**Figure 5:**
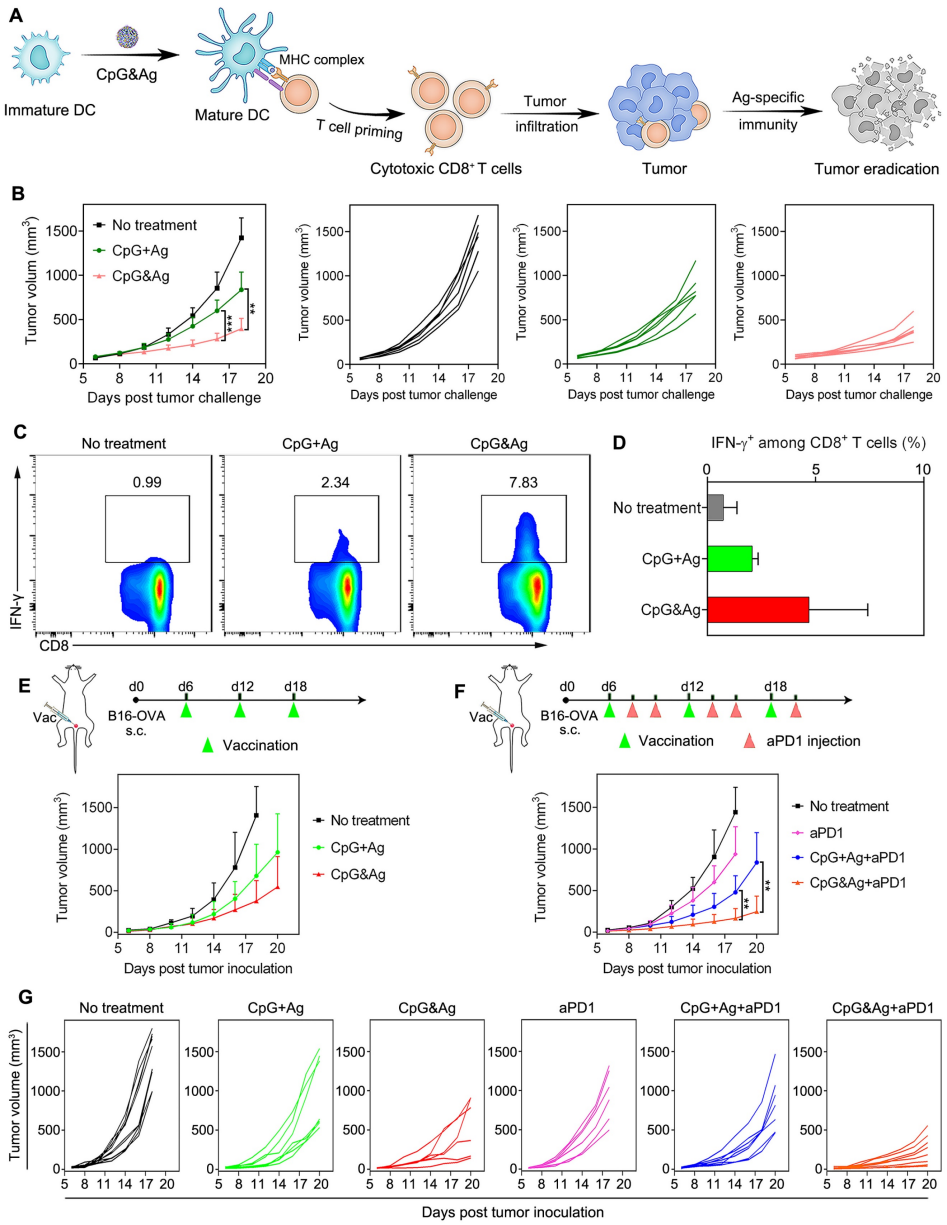
Antitumor immunotherapy of CpG&Ag (CpG: 3 nmol, Ag: ^P^SKRKKK(peg5)CSIINFEKL, 10 μg) combined with ICB on melanoma model. **(A)** Schematic showing the mechanism of CpG&Ag-based antitumor immunotherapy. **(B)** Tumor progression of pre-vaccinated C57BL/6 mice after challenged with B16-OVA cells through subcutaneous injection at right flank. **(C)** Flow cytometry analysis and **(D)** quantification of intracellular IFN-γ^+^CD8^+^ T cells in peripheral blood after indicated treatment. **(E)** The B16-OVA tumor-bearing mice were immunized with indicated vaccine formulations at indicated time. **(F)** The B16-OVA tumor-bearing mice were immunized with indicated vaccine formulations on day6, day12 and day18. On the 2nd and 4th day after each vaccination, the mice were intraperitoneally injected with aPD1 (200 μg per mouse). **(G)** The tumor growth in each group was monitored every other day. Data are presented as means ± s.d. (n = 5-8/group). **P < 0.01, ***P < 0.001.

Intrigued by the robust immunostimulation and efficient codelivery of antigen and CpG to APCs both *in vitro* and *in vivo*, we then challenged the C57BL/6 mice with B16-OVA cells through subcutaneous injection 7 days after the third vaccination (**Figure 5A** and **Figure S13A**). It revealed that the CpG&Ag (Ag: ^P^SKRKKK(peg5)CSIINFEKL) immunized mice had minimal tumor masses during the 18 days of tumor progression monitoring. While for mice immunized with CpG + Ag, the tumor growth was not effectively suppressed and no tumor regression was observed compared with nanovaccine group (**Figure 5B**). Moreover, no systemic toxicity was observed in comparison to control groups during the tumor challenge study (**Figure S13B and S14**). Flow cytometry analysis revealed that the vaccination with CpG&Ag (Ag: ^P^SKRKKK(peg5)CSIINFEKL) induced significant increase in polyfunctional IFN-γ^+^CD8^+^ T cells (7.83%), whereas only 2.34% cells were elicited by the free mixture formulation (**Figure 5C-D**). Antitumor immunotherapy study showed that the B16-OVA tumor growth was notably restrained compared with soluble vaccine formulation or no treatment groups (**Figure 5E**). Despite the effective tumor growth inhibition, no complete tumor eradication was observed in the nanovaccine treatment group, which could be attributed to the immunosuppressive tumor microenvironment. Flow cytometry analysis revealed the overexpression of both programmed cell death-1 (PD-1) and the ligand 1 (PD-L1) among CD8^+^ T cells and tumor cells, respectively (**Figure S15**). To block the immunosuppression mediated by PD-1/PD-L1 pathway, we combined the nanovaccine CpG&Ag with anti-PD-1 antibody (aPD1) for combinational immunotherapy. The combination with aPD1 further enhanced the tumor growth inhibition or regression, with ~50% of them significantly suppressed or completely regressed (**Figure 5F-G**). In contrast, no tumor eradication was observed in aPD1 or the combination with soluble vaccine formulation CpG + Ag + aPD1 group. Notably, no obvious body weight loss or systemic toxicity was detected during the immunotherapy study (**Figure S16** and **S17**).

**Figure 6:**
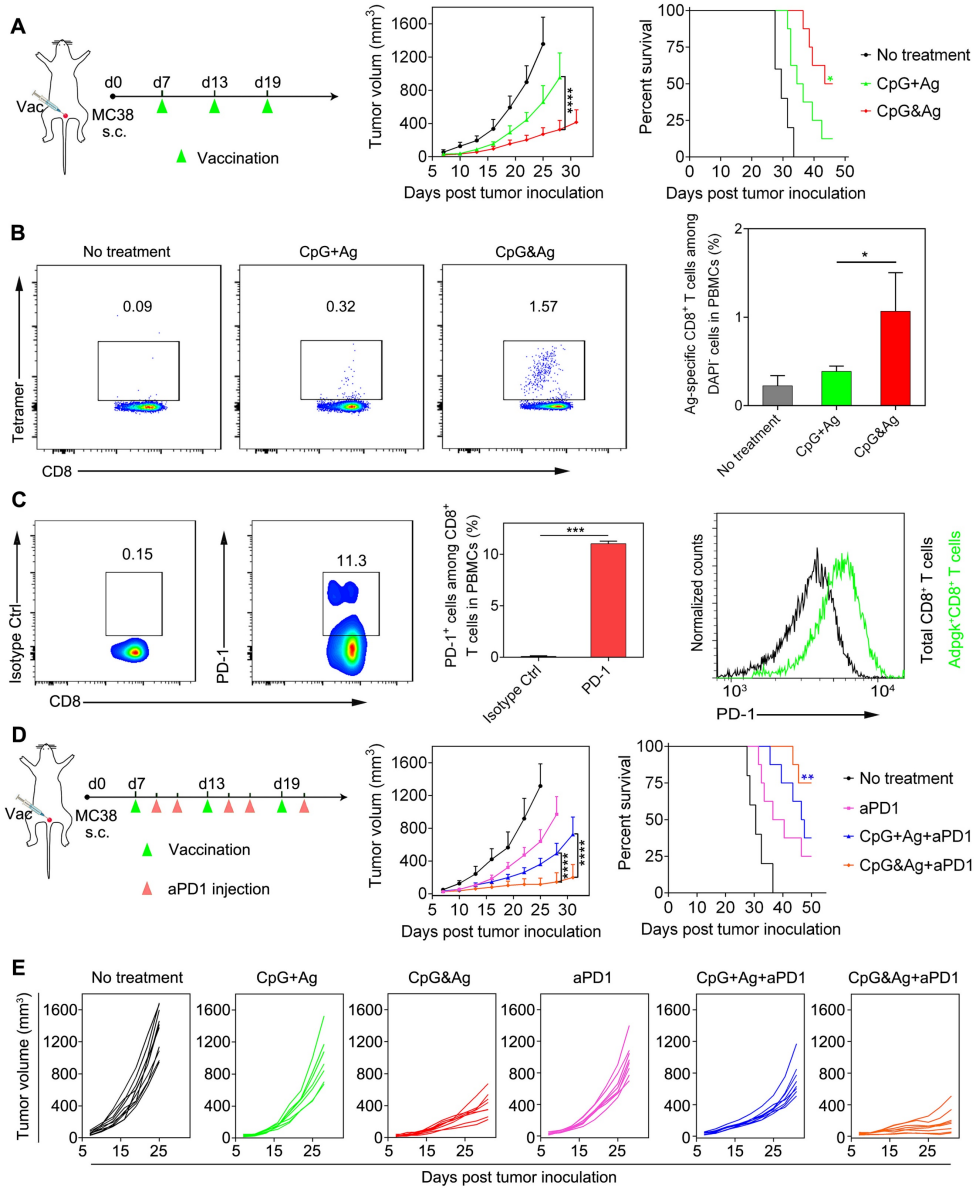
Neoantigen-specific antitumor immunotherapy of CpG&Ag (Ag: cp(peg)Adpgk) combined with ICB on MC-38 colon tumor model. **(A)** Left: MC-38 tumor-bearing mice were vaccinated with indicated vaccine formulations at indicated time. Middle: tumor growth of each group monitored every other day. Right: animal survival of the mice subjected to different treatments. **(B)** Flow cytometry scatter plots and the frequency of Ag-specific CD8^+^ T cells in peripheral blood collected from the mice 7 days after the third vaccination with indicated vaccine formulations (CpG: 3 nmol, Ag: cp(peg)Adpgk, 22 μg). **(C)** Flow cytometric evaluation of PD-1 expression on peripheral CD8^+^ T cells upon immunized with the CpG&Ag. (n = 3) **(D)** Left: The MC-38 tumor-bearing mice were vaccinated with indicated vaccine formulations on days 7, 13 and 19. On day 2 and day 4 after each vaccination, the mice were intraperitoneally injected with aPD1 (200 μg per mouse). Middle: tumor growth of each group monitored every other day. Right: animal survival of the mice subjected to different treatments. **(E)** The tumor growth in each group was monitored every other day. Data are presented as means ± s.d. (n = 5-8/group). ns not significant, *P < 0.05, ***P < 0.001, ****P < 0.0001.

To further explore the applications of our nanovaccine, MC-38 colon tumor model with Adpgk neoantigen mutation (ASMTN**R**EL → ASMTN**M**ELM) was employed to evaluate the Adpgk-specific antitumor immunotherapy. We immunized MC-38 tumor-bearing C57BL/6 mice with CpG&Ag or CpG + Ag (Ag: cp(peg)Adpgk) vaccine formulations (**Figure 6A**). Tumor growth monitoring demonstrated that the nanovaccine treatment led to dramatic inhibition of tumor progression and longer animal survival, compared with the CpG + Ag treatment or no treatment groups (**Figure 6A**). Flow cytometric analysis showed that the nanovaccine formulation elicited much higher propotion of Ag-specific CD8^+^ T cells than control groups after the treatment, demonstrating robust T-cell cross-priming induced by CpG&Ag (**Figure 6B** and **Figure S18**). Considering immunosuppression and T-cell exhaustion mediated by immune checkpoint such as PD-1/PD-L1 pathway during the antitumor immunotherapy based on the nanovaccine (**Figure 6C** and **Figure S19**), the aPD1 was leveraged for checkpoint blockade and combinational immunotherapy with the nanovaccine. The combination with PD-1 inhibitor led to significant tumor growth inhibition and tumor rejection in ~38% of the mice after the nanovaccine treatment (**Figure 6D** and **Figure S20**). However, the tumors of a fraction of tumor-bearing mice are not completely eradicated even in combination with PD-1 inhibitor. This may be attributed to the immunosuppression mediated by other signaling pathways such as cytotoxic T lymphocyte-associated molecule-4 (CTLA-4) and tumor heterogeneity 43-45. Additionally, prolonged animal survival was observed after the treatment, with ~75% of mice survived 50 days after the tumor inoculation (**Figure 6D-E**). In contrast, no tumor eradication was observed and only a survival rate of ~38% was measured in the soluble vaccine formulation CpG + Ag + aPD1 group (**Figure 6D-E**). Together, the engineered neoantigen nanovaccine CpG&Ag induced potent neoantigen-specific T-cell responses and tumor growth inhibition or even tumor rejection when combined with ICB therapy.

## Conclusion

In conclusion, we have disclosed a facile and general methodology for nano-assembly of neoantigen peptide and oligonucleotide therapeutics without the introduction of artificial carriers. The nanovaccine was engineered by programming the neoantigen peptide with a phosphorylated serine-functionalized short cationic module to enhance the interaction between neoantigen peptide, metal ions and CpG agonist 40,46, thus promoting the assembly of neoantigen and CpG into monodispersed nanoparticles. We demonstrated that the engineered nanovaccine CpG&Ag promoted the codelivery of neoantigen peptides and adjuvants to lymphoid organs with high efficiency, eliciting neoantigen-specific immune responses with negligible systemic toxicity. Furthermore, the immunogenicity of nanovaccine was profoundly potentiated when combined with immune checkpoint blockade therapy, leading to complete tumor regression of established B16-OVA and MC-38 tumor models. This programming and nano-assembling strategy allows efficient codelivery of peptide and oligonucleotide therapeutics without artificial carriers both *in vitro* and *in vivo*, which has great significance and potential for clinical applications.

## Materials and methods

### Materials

All peptides (**Table S1**) used in this study were synthesized by GL Biochem (Shanghai, China) and used as supplied without further purification. The oligonucleotides (**Table S1**) were synthesized and purified by Sangon Biotech Co. (Shanghai, China). Iron(II) chloride tetrahydrate (FeCl_2_·4H_2_O) was obtained from Sigma-Aldrich. Fetal bovine serum (FBS) was purchased from Gibco (CA, USA). LysoTracker Green DND-26 and Hoechst 33342 were purchased from Invitrogen. Anti-mouse CD3, anti-mouse PD-1-FITC, anti-mouse PD-L1-PE, anti-mouse SIINFEKL/H-2K^b^-PE monoclonal antibodies were acquired from eBioscience. Anti-mouse CD8a-PE, anti-mouse CD11c-FITC, anti-mouse CD80-PE, anti-mouse CD86-APC antibodies were purchased from Biolegend. OVA tetramer-APC was purchased from MBL. The ELISA kits for TNF-α, IL-6 and IL-12 analysis were purchased from Thermo Fisher Scientific. Anti-mouse PD-1 antibody was acquired from BioXcell. The mice used in this study were obtained from Charles River (Beijing, China).

### Instrumentations

The fluorescence spectra and UV-vis absorption spectra were recorded by a FS5 Spectrofluorometer (Edinburgh, UK) and Cary 5000 UV-Vis-NIR spectrophotometer (Agilent, USA) at room temperature, respectively. The nanoparticle size distribution and zeta potential were analyzed using a Litesizer 500 (Anton Paar, AT) at 25 ºC. Transmission electron microscopy (TEM) images were acquired on FEI Tecnai G2 Spirit electron microscope (FEI, USA). High-angle annular dark-field scanning TEM (HAADF-STEM) elemental mapping was performed on a JEOL JEM-2100F TEM (JEOL, Japan). The water used in this study was obtained from a Millipore ultrapure Milli-Q water system (Billerica, USA). Agarose gel electrophoresis images were taken using BioRad imaging system (Biorad, USA). Confocal laser scanning microscopy (CLSM) images were acquired using a Zeiss LSM710 confocal imaging system (Zeiss, Germany). Flow cytometry analysis was performed on a CytoFLEX LX flow cytometer (Beckman, USA). *In vivo* fluorescence images were acquired using an IVIS SPECTRUM *in vivo* imaging system (PerkinElmer, USA).

### Preparation of CpG&Ag nanovaccine

The CpG&Ag was prepared by respectively premixing cp(peg)Ag (80 μM, 300 μL) and CpG (50 μM, 300 μL) in aqueous solution with FeCl_2_·4H_2_O (10 mM, 5 μL) aqueous solution in two 1.5 mL EP tubes. Then the solutions in two tubes were thoroughly mixed in one EP tube and vortexed for 10 s and incubated at 95 ℃ for 2 h without disturbing. After natural cooling, the resulting solution was washed with MQ water by centrifugating at 15000 g for 10 min. The obtained CpG&Ag was redispersed in MQ water and kept at 4 ℃ for further use. To investigate the impact of cp(peg)Ag on nanoparticle formation, different molar ratios of cp(peg)Ag:CpG with the concentration of CpG maintained at 25 μM were supplemented for nanovaccine preparation.

### Loading efficiency and biostability evaluation

To investigate the loading efficiency of neoantigen and CpG, the supernatants and washing solutions were collected. The CpG concentration was determined according to the absorption at 260 nm measured by UV-vis spectrometry. Then the loading efficiency of CpG could be calculated using this formula: LE= ((A^t^-A^s^)/A^t^) × 100 % where A^t^ is the total amount of CpG added during self-assembly, and A^s^ denotes the amount of CpG in supernatant. The neoantigen (FITC-labeled) concentration in the supernatant was determined by fluorescence spectroscopy and the loading efficiency was calculated using the above formula. For stability evaluation, the prepared nanovaccine was incubated in HEPES buffer (PH 7.4) at 37 °C with gentle shaking for different time durations, followed by TEM imaging and size distribution analysis using a Litesizer 500.

### Agarose gel electrophoresis

The successful loading of neoantigen was confirmed using agarose gel electrophoresis (1% agarose). The prepared CpG&Ag (Ag: FITC-labeled) was premixed with DNA loading buffer (6 ×, Invitrogen), then loaded into the agarose gel matrix in 1 × TAE running buffer and run at 120 V for 45 min. After electrophoresis, the gel FireRed and fluorescence images were respectively acquired using a BioRad imaging system.

### Cell culture and cytotoxicity assay

DC2.4 cells were cultured in RPMI 1640 culture medium supplemented with 10% FBS and 1% penicillin/streptomycin. RAW264.7 macrophages, B16-OVA and MC38 cells were cultured in DMEM culture medium supplemented with 10% FBS and 1% penicillin/streptomycin. The cells were maintained in a humidified incubator containing 5% CO_2_ at 37 ℃. For cytotoxicity evaluation, DC2.4 cells were seeded in a 96-well plate and cultured for 24 h to reach 60% confluence before experiment. Then the medium was replaced with CpG&Ag (Ag: cp(peg)CSIINFEKL)-supplemented culture medium at different concentrations. After 24h incubation, the medium was discarded and the fresh cell culture medium supplemented with 10% MTT solution was added. After another 2 h incubation in the humidified incubator and replace the culture medium with DMSO, the absorbance at 570 nm was measured using a microplate reader (ThermoFisher Scientific).

### Confocal fluorescence imaging

DC2.4 cells or RAW264.7 macrophages (1 × 10^5^) were seeded in confocal dishes and cultured for 24 h before experiment. Then the cells were treated with CpG + Ag and CpG&Ag (Ag: cp(peg)CSIINFEK_(FITC)_L) (CpG: 250 nM, Ag: 380 nM) supplemented in fresh culture medium. After incubated for indicated time durations, the nuclei and lysosomes of the cells were respectively stained with Hoechst 33342 and LysoTracker Red. Then the cells were washed 3 times with PBS and replenished with 100 mL PBS for confocal fluorescence imaging.

### Flow cytometry analysis

For flow cytometry analysis, DC2.4 cells were seeded in 24-well plates (5 × 10^4^ cells/well) and cultured for 24 h before the assay. The cells were treated with Ag, CpG + Ag or CpG&Ag (Ag: cp(peg)CSIINFEK_(FITC)_L) (CpG: 250 nM, Ag: 380 nM) for indicated time as the procedure described above. After the treatment, the cells were incubated with anti-SIINFEKL/H-2K^b^-PE monoclonal antibody or anti-CD80/86 antibody, then the cells were collected and resuspended in PBS for flow cytometry analysis. The level of proinflammatory factors including TNF-α, IL-6 and IL-12 secreted from DCs after indicated treatments were measured by ELISA kits.

### LN draining of CpG&Ag and elicitation of Ag-specific T cell responses

All animal studies were performed according to the guidelines of Institutional Animal Care and Use Committee (IACUC) of Hangzhou Medical College Laboratory Animal Center (ZJCLA-IACUC-20010298). For LN draining assays, female C57BL/6 mice (6-8 weeks) were subcutaneously injected with CpG + Ag or CpG&Ag (CpG: Cy5 labeled, 1 nmol per mouse; Ag: cp(peg)CSIINFEK_(FITC)_L, 3.5 μg per mouse) at the tail base. 12 h post-injection, the inguinal dLNs were harvested, then Cy5 and FITC fluorescence images were respectively acquired using an IVIS Spectrum *in vivo* imaging system. After imaging, the LNs were dissociated into single cells using DNase I and collagenase according to the manufacturer's instructions. Then cells were collected and stained with anti-mouse CD11c antibody for flow cytometric analysis of Cy5 and FITC signals in DCs. For elicitation of neoantigen-specific T cell responses analysis, female C57BL/6 mice (6-8 weeks) were vaccinated with indicated vaccine formulations on days 0, 7, and 14 (CpG: 3 nmol, Ag: ^P^SKRKKK(peg5)CSIINFEKL, 10 μg), then peripheral blood was collected for Ag-specific CD8^+^ T cells frequency evaluation on day 21. The red blood cells were lysed with lyse buffer for 3 to 5 min, followed by centrifugation at 1500 x g for 5 min and remove the supernatant. Then the cells were washed with PBSA buffer (PBS supplemented with 1% w/v BSA) and incubated at room temperature. After stained with Live/Dead Cell Staining Kit and blocked with anti-CD16/CD32, the cells were further stained with antibody cocktail (mouse CD8-FITC antibody, OVA tetramer-APC antibody). Then the cells were washed two times with PBSA buffer and resuspended in the buffer for flow cytometry analysis.

### Investigation of T-cell immunity and PD-1/PD-L1 expression

To evaluate the expression levels of PD-L1 and PD-1 on tumor cells and PBMCs after the nanovaccine treatment, the blood was collected on day 21 and the tumors were collected at the end of experiment for flow cytometry analysis. For PBMCs staining, the blood was centrifuged for blood cells enrichment and treated with red blood cell lysis buffer. After centrifugation, the collected cells were washed with staining buffer (PBS supplemented with 1% FBS), and stained with Live/Dead Cell Staining Kit. After blocked with anti-CD16/CD32, the cells were further stained with antibody cocktail (mouse CD3-APC antibody, mouse CD8-FITC antibody, mouse PD1-PE antibody). To analyze the PD-L1 expression on tumor cells, the harvested tissues were cut into pieces and lysed in disassociation buffer (1 mg/mL of collagenase IV and 200U/mL of DNase I in 5% FBS buffered RPMI buffer) at 37 ℃for 30 min. Then the suspension was filtered through a 100 μm strainer, washed with staining buffer and stained with antibody cocktail (mouse CD3-APC antibody, mouse CD8-FITC antibody, mouse PD-L1-PE antibody). Then the cells were washed with staining buffer for flow cytometry analysis.

### *In vivo* cancer immunotherapy

For tumor challenge study, the immunized mice were challenged with 1.5 × 10^5^ B16-OVA cells per mouse on the right shoulder at day 7 after the last vaccination. The tumor growth was monitored every other day and calculated using the following formula: V = length × width × width/2. For xenograft tumor models construction, female C57BL/6 mice (6-8 weeks) were subcutaneously injected with B16-OVA or MC38 cells (2 × 10^5^ cells/100 μL PBS) on their right shoulder. The tumor sizes and body weight were measured at indicated time points and volumes were calculated using the above formula. When tumor volume reached ~60 mm^3^, the mice were randomly divided into 6 groups with 5-8 mice in each group for the immunotherapy study. The mice in 5 groups were subcutaneously injected with CpG + Ag, CpG&Ag, aPD1, CpG + Ag + aPD1, CpG&Ag + aPD1 (CpG: 3 nmol per mouse; Ag, ^P^SKRKKK(peg5)CSIINFEKL: 10 μg per mouse; Ag, cp(peg)Adpgk: 22 μg per mouse; aPD1: 200 μg per mouse) at the tail base on the indicated day. For combinational aPD1 immune checkpoint blockade therapy, aPD1 was intraperitoneally administered at the indicated time points. The tumor growth and mice body weight were measured at indicated time and tumor volumes were calculated according to the formula described above throughout the therapy study. The mice were euthanized and main organs were harvested for H&E staining analysis at the end of immunotherapy.

### Statistical analysis

The number of cells and animal groups is included in figure legends. Statistical difference comparison between different groups was analyzed using one-way analysis of variance (ANOVA) or unpaired Student's t test in GraphPad Prism software. ns, not significant, *P < 0.05, **P < 0.01, ***P < 0.001, and ****P < 0.0001. All values in the manuscript were presented as means ± s.d.

## Supplementary Material

Supplementary materials and methods, figures.

## Abbreviations

APCs: antigen-presenting cells
TLR9: toll-like receptor 9
CAR-T: chimeric antigen receptor T
ICIs: immune checkpoint inhibitors
cp: cationic module
CpG&Ag: nanovaccine co-assembled of programmed neoantigen peptide and CpG
CpG-Fe: nanoparticles prepared by assembly of CpG and Fe^2+^
HAADF-STEM: high-angle annular dark-field scanning transmission electron microscopy
TNF-α: tumor necrosis factor-α
dLN: draining lymph node
aPD1: anti-PD-1 antibody
^P^S: phosphorylated serine
DLS: Dynamic light scattering
