# Supplementary material for: Programming peptide-oligonucleotide nano-assembly for engineering of neoantigen vaccine with potent immunogenicity
Source: Theranostics. 2024 Mar 17;14(6):2290–303. doi: 10.7150/thno.93395 (PMC11024849; doi:10.7150/thno.93395)
Supplement: Supplementary file 1 — Supplementary materials and methods, figures. [file thnov14p2290s1.pdf]

Supporting information

Programming peptide-oligonucleotide nano-assembly for engineering of neoantigen vaccine with potent immunogenicity

Zhichu Xiang[1,2], Jianhua Lu[1], Shangrui Rao[1], Chenxing Fu[6], Yuying Yao[6], Yongdong Yi[1], Yang Ming[2], Weijian Sun[1*], Weisheng Guo[6*], Xiaoyuan Chen[2,3,4,5*]

[1]Department of Gastrointestinal Surgery, The Second Affiliated Hospital and Yuying Children's Hospital of Wenzhou Medical University, Wenzhou 325027, China. fame198288@126.com

[2]Departments of Diagnostic Radiology, Surgery, Chemical and Biomolecular Engineering, and Biomedical Engineering, Yong Loo Lin School of Medicine and College of Design and Engineering, National University of Singapore, Singapore 119074, Singapore. chen.shawn@nus.edu.sg.

[3]Clinical Imaging Research Centre, Centre for Translational Medicine, Yong Loo Lin School of Medicine, National University of Singapore, Singapore 117599, Singapore.

[4]Nanomedicine Translational Research Program, Yong Loo Lin School of Medicine, National University of Singapore, Singapore 117597, Singapore.

[5]Institute of Molecular and Cell Biology, Agency for Science, Technology, and Research (A*STAR), 61 Biopolis Drive, Proteos, Singapore 138673, Singapore.

[6]Department of Minimally Invasive Interventional Radiology, the State Key Laboratory of Respiratory Disease, School of Biomedical Engineering & The Second Affiliated Hospital, Guangzhou Medical University, Guangzhou, 510260, China. tjuguoweisheng@126.com.

Materials and methods

Materials. All peptides (Table S1) used in this study were synthesized by GL Biochem (Shanghai, China) and used as supplied without further purification. The oligonucleotides (Table S1) were synthesized and purified by Sangon Biotech Co. (Shanghai, China). Iron(II) chloride tetrahydrate ($FeCl_2 \cdot 4H_2O$) was obtained from Sigma-Aldrich. Fetal bovine serum (FBS) was purchased from Gibco (CA, USA). LysoTracker Green DND-26 and Hoechst 33342 were purchased from Invitrogen. Anti-mouse CD3, anti-mouse PD-1-FITC, anti-mouse PD-L1-PE, anti-mouse SIINFEKL/H-2K^b-PE monoclonal antibodies were acquired from eBioscience. Anti-mouse CD8a-PE, anti-mouse CD11c-FITC, anti-mouse CD80-PE, anti-mouse CD86-APC antibodies were purchased from Biolegend. OVA tetramer-APC was purchased from MBL. The ELISA kits for TNF-α, IL-6 and IL-12 analysis were purchased from Thermo Fisher Scientific. Anti-mouse PD-1 antibody was acquired from BioXcell. The mice used in this study were obtained from Charles River (Beijing, China).

Instrumentations. The fluorescence spectra and UV-vis absorption spectra were recorded by a FS5 Spectrofluorometer (Edinburgh, UK) and Cary 5000 UV-Vis-NIR spectrophotometer (Agilent, USA) at room temperature, respectively. The nanoparticle size distribution and zeta potential were analyzed using a Litesizer 500 (Anton Paar, AT) at 25 ºC. Transmission electron microscopy (TEM) images were acquired on FEI Tecnai G2 Spirit electron microscope (FEI, USA). High-angle annular dark-field scanning TEM (HAADF-STEM) elemental mapping was performed on a JEOL JEM-2100F TEM (JEOL, Japan). The water used in this study was obtained from a Millipore ultrapure Milli-Q water system (Billerica, USA). Agarose gel electrophoresis images were taken using BioRad imaging system (Biorad, USA). Confocal laser scanning microscopy (CLSM) images were acquired using a Zeiss LSM710 confocal imaging system (Zeiss, Germany). Flow cytometry analysis was performed on a CytoFLEX LX flow cytometer (Beckman, USA). *In vivo* fluorescence images were acquired using an IVIS SPECTRUM *in vivo* imaging system (PerkinElmer, USA).

Preparation of CpG&Ag nanovaccine. The CpG&Ag was prepared by respectively premixing cp(peg)Ag (80 μM, 300 μL) and CpG (50 μM, 300 μL) in aqueous solution with $FeCl_2 \cdot 4H_2O$ (10 mM, 5 μL) aqueous solution in two 1.5 mL EP tubes. Then the solutions in two tubes were thoroughly mixed in one EP tube and vortexed for 10 s and incubated at

95 °C for 2 h without disturbing. After natural cooling, the resulting solution was washed with MQ water by centrifugating at 15000 g for 10 min. The obtained CpG&Ag was redispersed in MQ water and kept at 4 °C for further use. To investigate the impact of cp(peg)Ag on nanoparticle formation, different molar ratios of cp(peg)Ag:CpG with the concentration of CpG maintained at 25 μM were supplemented for nanovaccine preparation.

Loading efficiency and biostability evaluation. To investigate the loading efficiency of neoantigen and CpG, the supernatants and washing solutions were collected. The CpG concentration was determined according to the absorption at 260 nm measured by UV-vis spectrometry. Then the loading efficiency of CpG could be calculated using this formula: LE= $((A^t-A^s)/A^t) \times 100$ % where A^t is the total amount of CpG added during self-assembly, and A^s denotes the amount of CpG in supernatant. The neoantigen (FITC-labelled) concentration in the supernatant was determined by fluorescence spectroscopy and the loading efficiency was calculated using the above formula. For stability evaluation, the prepared nanovaccine was incubated in HEPES buffer (PH 7.4) at 37 °C with gentle shaking for different time durations, followed by TEM imaging and size distribution analysis using a Litesizer 500.

Agarose gel electrophoresis. The successful loading of neoantigen was confirmed using agarose gel electrophoresis (1% agarose). The prepared CpG&Ag (Ag: FITC-labelled) was premixed with DNA loading buffer (6 ×, Invitrogen), then loaded into the agarose gel matrix in 1 × TAE running buffer and run at 120 V for 45 min. After electrophoresis, the gel FireRed and fluorescence images were respectively acquired using a BioRad imaging system.

Cell culture and cytotoxicity assay. DC2.4 cells were cultured in RPMI 1640 culture medium supplemented with 10% FBS and 1% penicillin/streptomycin. RAW264.7 macrophages, B16-OVA and MC38 cells were cultured in DMEM culture medium supplemented with 10% FBS and 1% penicillin/streptomycin. The cells were maintained in a humidified incubator containing 5% CO_2 at 37 °C. For cytotoxicity evaluation, DC2.4 cells were seeded in a 96-well plate and cultured for 24 h to reach 60% confluence before experiment. Then the medium was replaced with CpG&Ag (Ag: cp(peg)CSIINFEKL)-supplemented culture medium at different concentrations. After 24h incubation, the

medium was discarded and the fresh cell culture medium supplemented with 10% MTT solution was added. After another 2 h incubation in the humidified incubator and replace the culture medium with DMSO, the absorbance at 570 nm was measured using a microplate reader (ThermoFisher Scientific).

Confocal fluorescence imaging. DC2.4 cells or RAW264.7 macrophages (1×10^5) were seeded in confocal dishes and cultured for 24 h before experiment. Then the cells were treated with CpG + Ag and CpG&Ag (Ag: cp(peg)CSIINFEK$_{(FITC)}$L) (CpG: 250 nM, Ag: 380 nM) supplemented in fresh culture medium. After incubated for indicated time durations, the nuclei and lysosomes of the cells were respectively stained with Hoechst 33342 and LysoTracker Red. Then the cells were washed 3 times with PBS and replenished with 100 mL PBS for confocal fluorescence imaging.

Flow cytometry analysis. For flow cytometry analysis, DC2.4 cells were seeded in 24-well plates (5×10^4 cells/well) and cultured for 24 h before the assay. The cells were treated with Ag, CpG + Ag or CpG&Ag (Ag: cp(peg)CSIINFEK$_{(FITC)}$L) (CpG: 250 nM, Ag: 380 nM) for indicated time as the procedure described above. After the treatment, the cells were incubated with anti-SIINFEKL/H-2K^b-PE monoclonal antibody or anti-CD80/86 antibody, then the cells were collected and resuspended in PBS for flow cytometry analysis. The level of proinflammatory factors including TNF-α, IL-6 and IL-12 secreted from DCs after indicated treatments were measured by ELISA kits.

LN draining of CpG&Ag and elicitation of Ag-specific T cell responses. All animal studies were performed according to the guidelines of Institutional Animal Care and Use Committee (IACUC) of Hangzhou Medical College Laboratory Animal Center (ZJCLA-IACUC-20010298). For LN draining assays, female C57BL/6 mice (6-8 weeks) were subcutaneously injected with CpG + Ag or CpG&Ag (CpG: Cy5 labelled, 1 nmol per mouse; Ag: cp(peg)CSIINFEK$_{(FITC)}$L, 3.5 μg per mouse) at the tail base. 12 h post-injection, the inguinal dLNs were harvested, then Cy5 and FITC fluorescence images were respectively acquired using an IVIS Spectrum in vivo imaging system. After imaging, the LNs were dissociated into single cells using DNase I and collagenase according to the manufacturer's instructions. Then cells were collected and stained with anti-mouse CD11c antibody for flow cytometric analysis of Cy5 and FITC signals in DCs. For elicitation of neoantigen-specific T cell responses analysis, female C57BL/6 mice (6-8 weeks) were

vaccinated with indicated vaccine formulations on days 0, 7, and 14 (CpG: 3 nmol, Ag: ^{P}SKRKKK(peg5)CSIINFEKL, 10 μg), then peripheral blood was collected for Ag-specific $CD8^+$ T cells frequency evaluation on day 21. The red blood cells were lysed with lyse buffer for 3 to 5 min, followed by centrifugation at 1500 x g for 5 min and remove the supernatant. Then the cells were washed with PBSA buffer (PBS supplemented with 1% w/v BSA) and incubated at room temperature. After stained with Live/Dead Cell Staining Kit and blocked with anti-CD16/CD32, the cells were further stained with antibody cocktail (mouse CD8-FITC antibody, OVA tetramer-APC antibody). Then the cells were washed two times with PBSA buffer and resuspended in the buffer for flow cytometry analysis.

Investigation of T-cell immunity and PD-1/PD-L1 expression. To evaluate the expression levels of PD-L1 and PD-1 on tumor cells and PBMCs after the nanovaccine treatment, the blood was collected on day 21 and the tumors were collected at the end of experiment for flow cytometry analysis. For PBMCs staining, the blood was centrifuged for blood cells enrichment and treated with red blood cell lysis buffer. After centrifugation, the collected cells were washed with staining buffer (PBS supplemented with 1% FBS), and stained with Live/Dead Cell Staining Kit. After blocked with anti-CD16/CD32, the cells were further stained with antibody cocktail (mouse CD3-APC antibody, mouse CD8-FITC antibody, mouse PD1-PE antibody). To analyze the PD-L1 expression on tumor cells, the harvested tissues were cut into pieces and lysed in disassociation buffer (1 mg/mL of collagenase IV and 200U/mL of DNase I in 5% FBS buffered RPMI buffer) at 37 °Cfor 30 min. Then the suspension was filtered through a 100 μm strainer, washed with staining buffer and stained with antibody cocktail (mouse CD3-APC antibody, mouse CD8-FITC antibody, mouse PD-L1-PE antibody). Then the cells were washed with staining buffer for flow cytometry analysis.

***In vivo* cancer immunotherapy.** For tumor challenge study, the immunized mice were challenged with 1.5×10^5 B16-OVA cells per mouse on the right shoulder at day 7 after the last vaccination. The tumor growth was monitored every other day and calculated using the following formula: V = length × width × width/2. For xenograft tumor models construction, female C57BL/6 mice (6-8 weeks) were subcutaneously injected with B16-OVA or MC38 cells (2×10^5 cells/100 μL PBS) on their right shoulder. The tumor sizes

and body weight were measured at indicated time points and volumes were calculated using the above formula. When tumor volume reached ~60 mm^3, the mice were randomly divided into 6 groups with 5-8 mice in each group for the immunotherapy study. The mice in 5 groups were subcutaneously injected with CpG + Ag, CpG&Ag, aPD1, CpG + Ag + aPD1, CpG&Ag + aPD1 (CpG: 3 nmol per mouse; Ag, ^{P}SKRKKK(peg5)CSIINFEKL: 10 μg per mouse; Ag, cp(peg)Adpgk: 22 μg per mouse; aPD1: 200 μg per mouse) at the tail base on the indicated day. For combinational aPD1 immune checkpoint blockade therapy, aPD1 was intraperitoneally administered at the indicated time points. The tumor growth and mice body weight were measured at indicated time and tumor volumes were calculated according to the formula described above throughout the therapy study. The mice were euthanized and main organs were harvested for H&E staining analysis at the end of immunotherapy.

Statistical Analysis. The number of cells and animal groups is included in figure legends. Statistical difference comparison between different groups was analyzed using one-way analysis of variance (ANOVA) or unpaired Student's t test in GraphPad Prism software. ns, not significant, *$P < 0.05$, **$P < 0.01$, ***$P < 0.001$, and ****$P < 0.0001$. All values in the manuscript were presented as means ± s.d.

Table S1. The sequences of DNA and peptide used in this study.

Name	Sequence (5'-3' or N-C terminus)
CpG	TCCATGACGTTCCTGACGTT
CpG (Cy5 labelled)	TCCATGACGTTCCTGACGTT-Cy5
cp(peg)OVA	^{P}SKRKKK(peg5)CSIINFEKL
cp(peg)OVA (FITC labelled)	^{P}SKRKKK(peg5)CSIINFEK$_{(FITC)}$L
cp(peg)Adpgk	^{P}SKRKKKKKK(peg5)CGIPVHLELASMTNMELMSSIVHQQVFPT

^{P}S denotes phosphorylated serine.

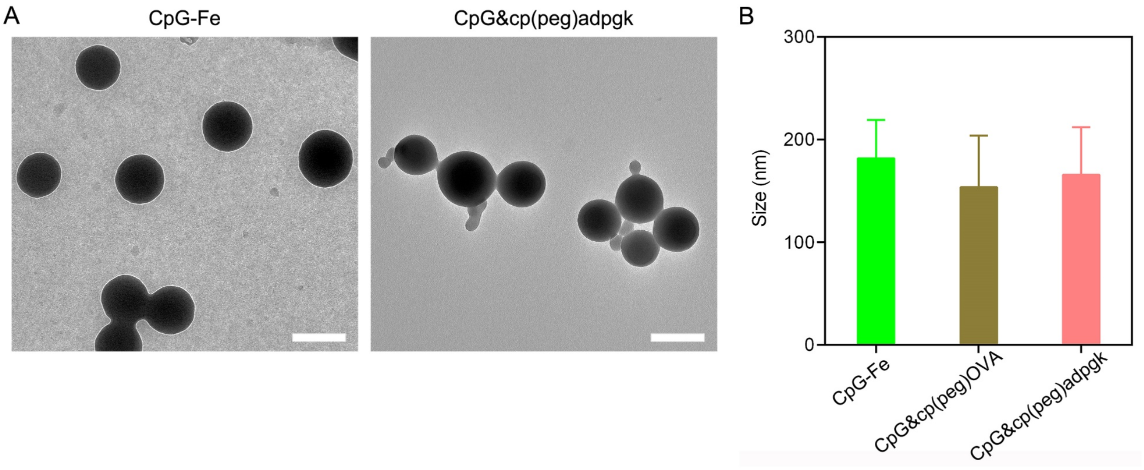

Figure S1. TEM images of (A) CpG-Fe and CpG&cp(peg)Adpgk. (B) Hydrodynamic size distribution of CpG-Fe, CpG&cp(peg)OVA and CpG&cp(peg)Adpgk. Scale bars, 200 nm. Data are presented as means ± s.d. (n=3).

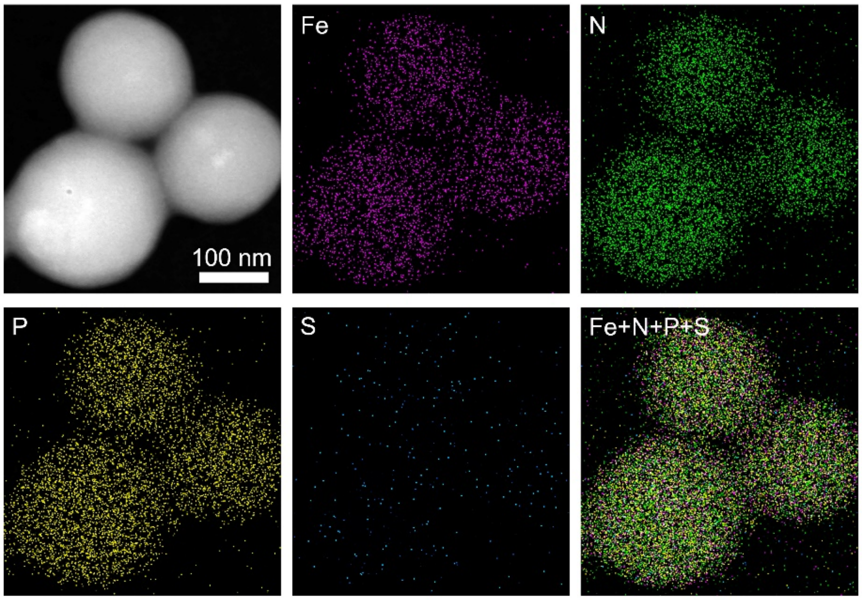

Figure S2. HAADF-STEM and elemental mapping images of CpG-Fe.

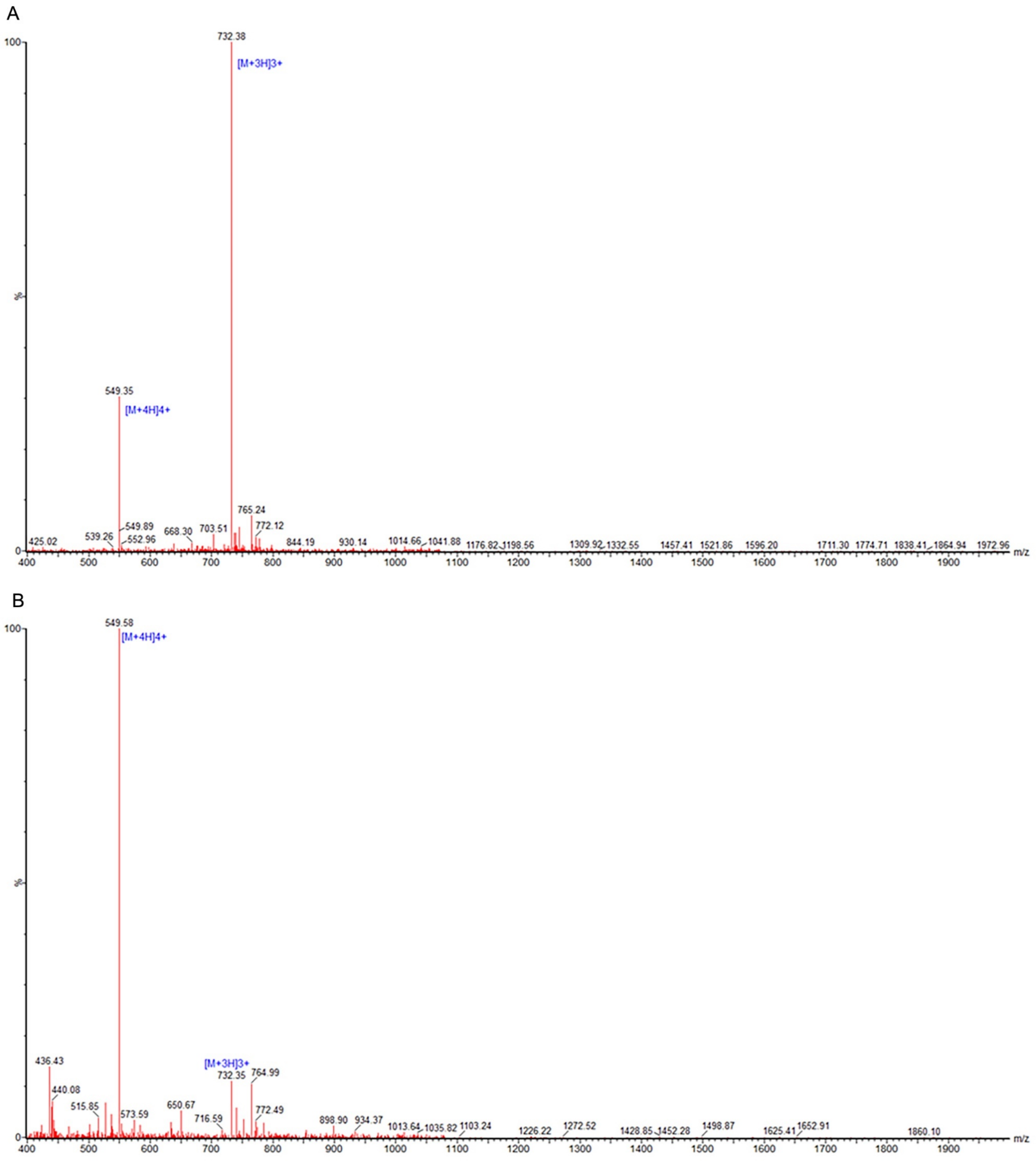

Figure S3. ESI-MS characterization of Ag: cp(peg)OVA sequence (A) before and (B) after treatment at 95°C.

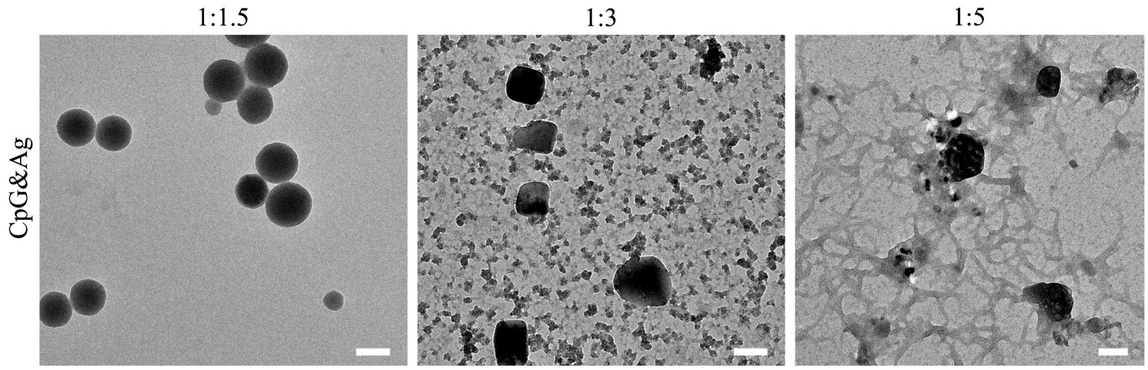

Figure S4. TEM images of CpG&Ag engineered with varying CpG:Ag (Ag: cp(peg)OVA) ratios. Scale bars, 100 nm.

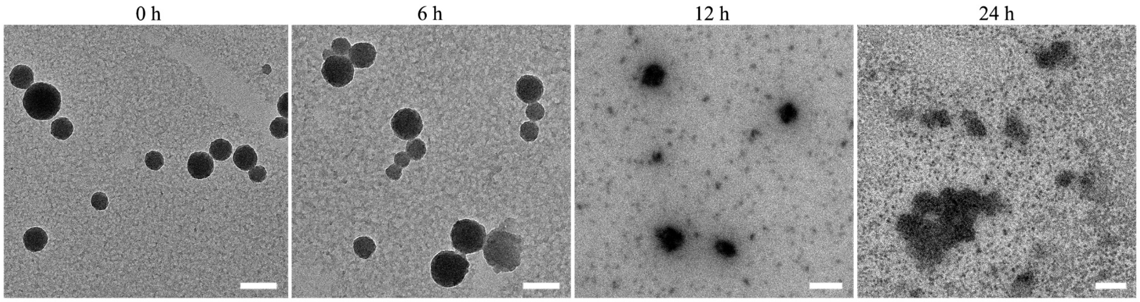

Figure S5. TEM images of CpG&cp(peg)OVA after incubated in HEPES buffer (pH 7.4) for different time. Scale bars, 200 nm.

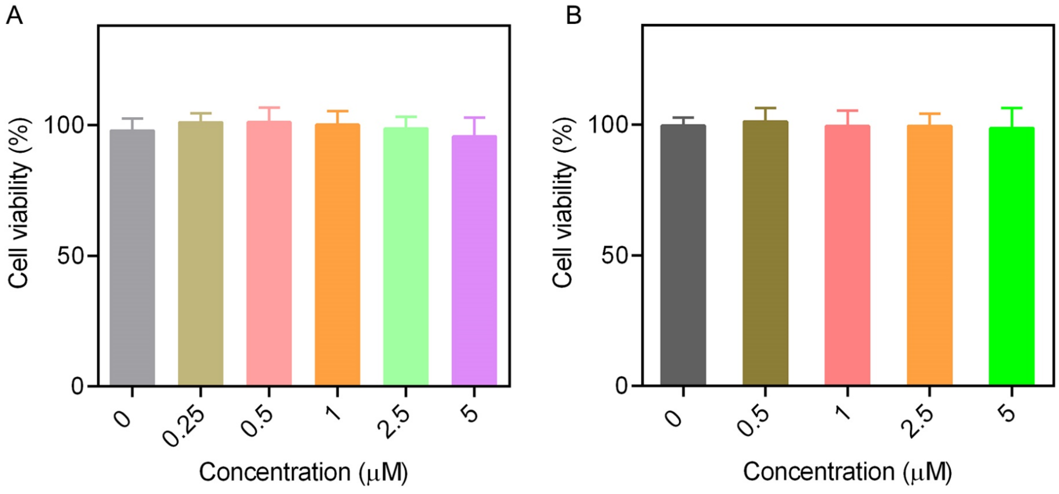

Figure S6. Cell viability of (A) DC2.4 cells and (B) macrophages after treated with CpG&cp(peg)OVA at different concentrations of CpG. Data are presented as means ± s.d. (n = 3).

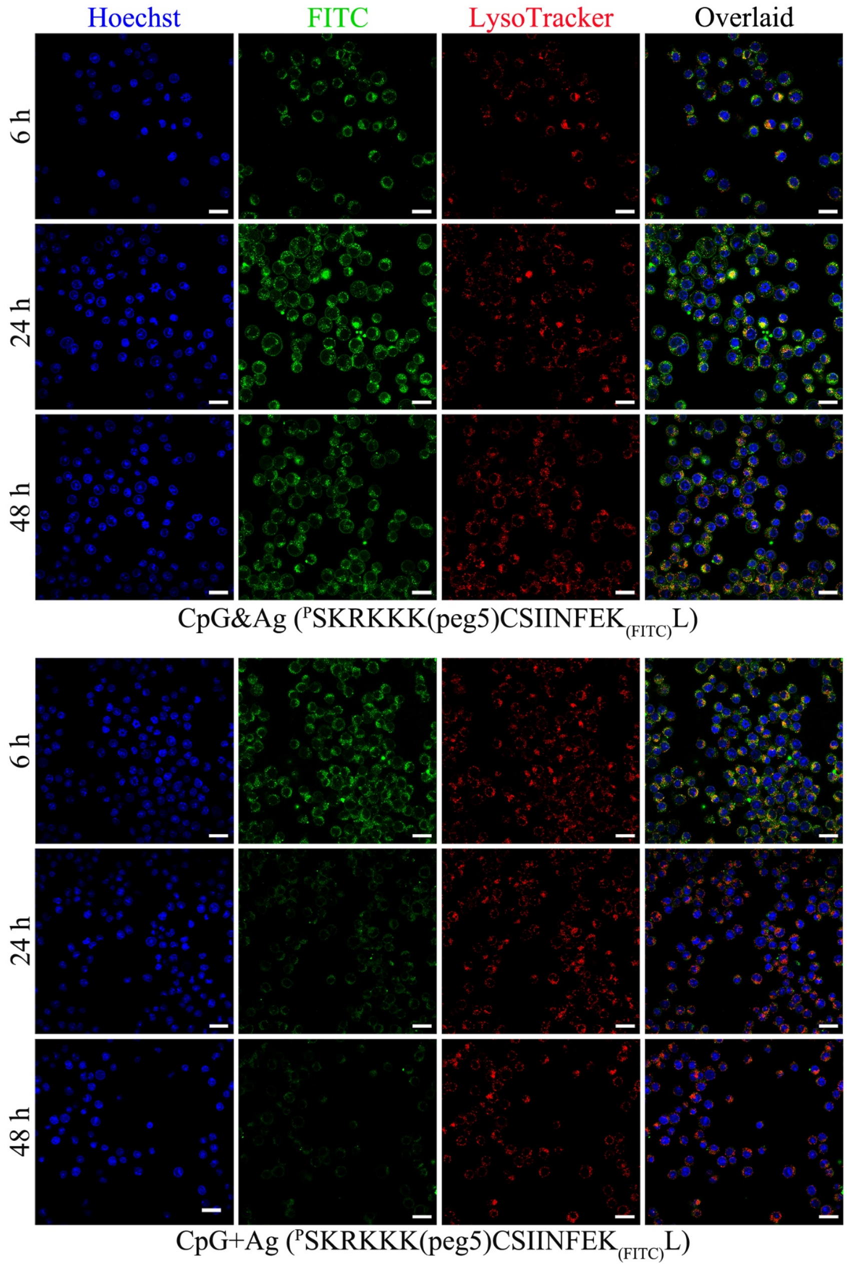

Figure S7. Confocal fluorescence images of DC2.4 cells after treated with CpG&Ag or CpG + Ag (Ag: ^{P}SKRKKK(peg5)CSIINFEK$_{(FITC)}$L) for 4 h, 24 h and 48 h (CpG: 250 nM, Ag: 380 nM). Scale bars, 20 μm.

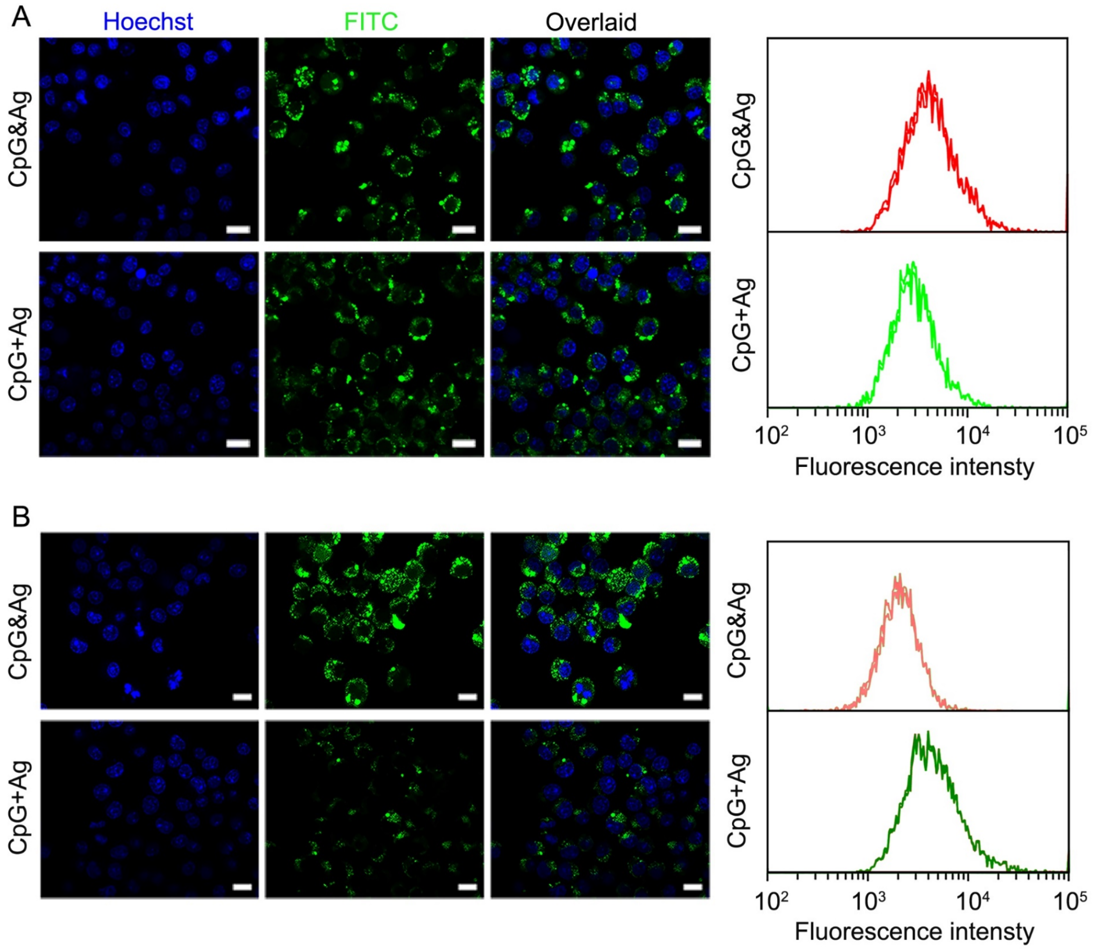

Figure S8. Confocal fluorescence images of RAW264.7 macrophages and corresponding fluorescence intensity quantification using flow cytometry after treated with CpG + Ag or CpG&Ag (Ag: ^{P}SKRKKK(peg5)CSIINFEK$_{(FITC)}$L) for (A) 6 h, and (B) 24 h (CpG: 250 nM, Ag: 380 nM). Scale bars, 20 μm.

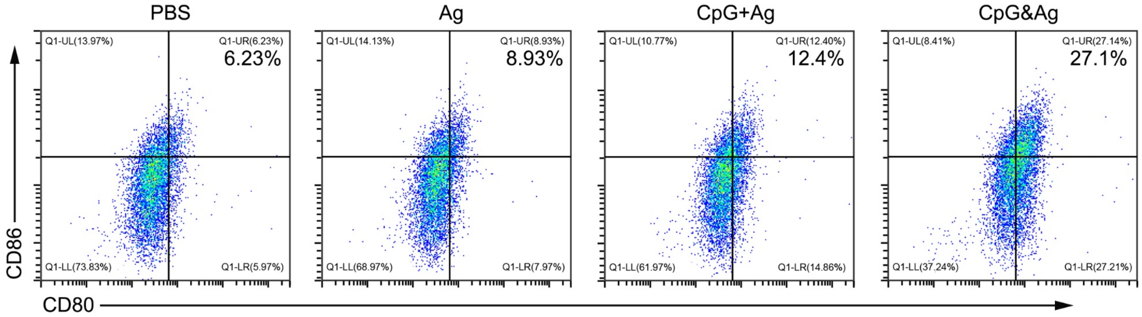

Figure S9. Flow cytometry analysis of costimulatory factors CD80 and CD86 expression on DC2.4 cells after indicated treatment.

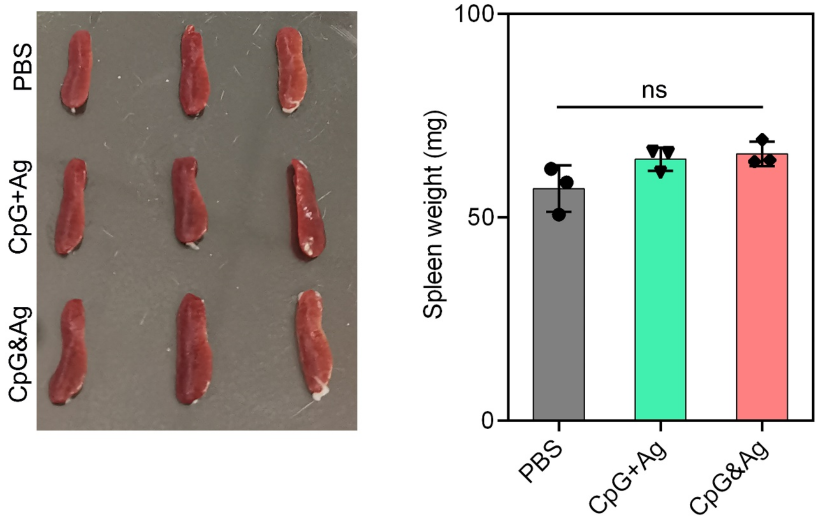

Figure S10. Images and weight of spleens collected from the mice on day 7 after the vaccination on day 0, 3, and 5. Data are presented as means ± s.d. (n = 3). ns not significant.

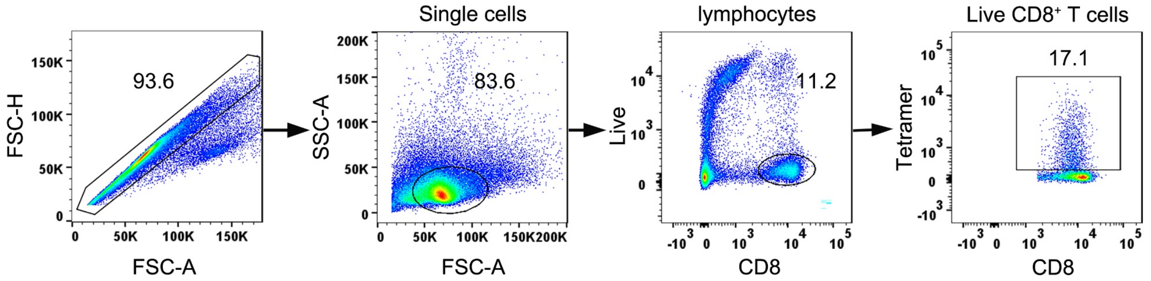

Figure S11. Gating tree exploited for flow cytometric evaluation of Ag-specific $CD8^+$ T cells in tetramer staining analysis.

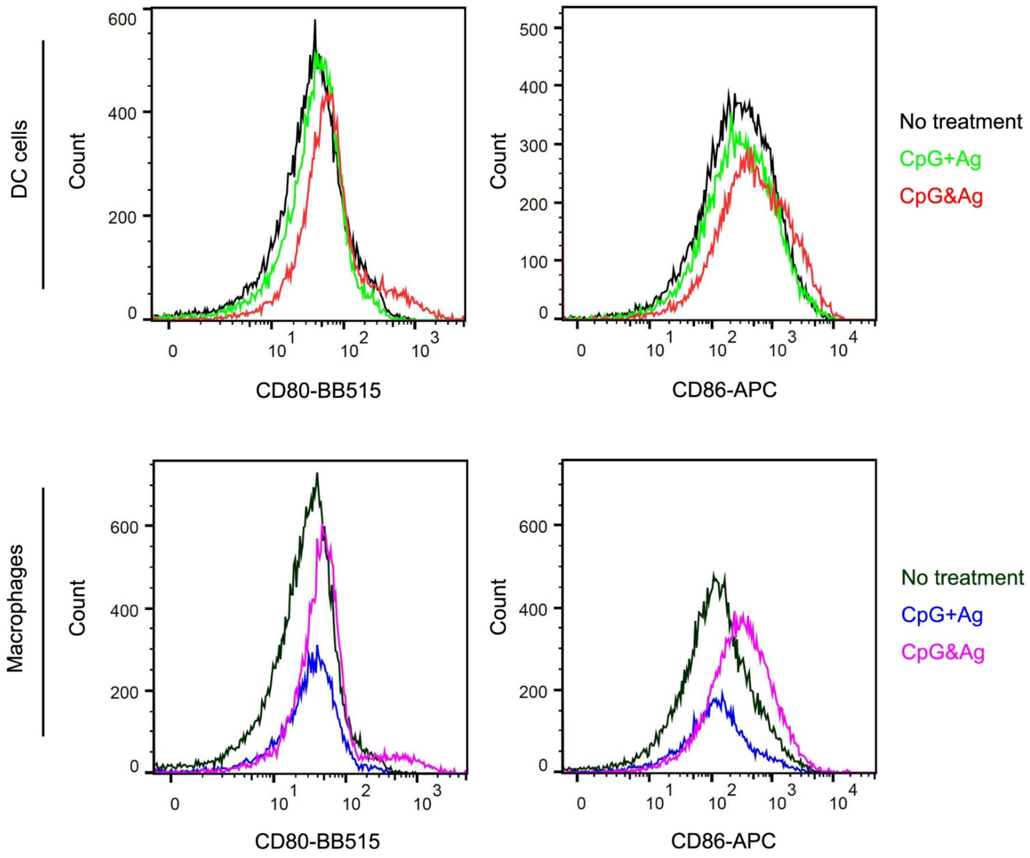

Figure S12. Flow cytometry analysis showed the increased CD80 and CD86 expression in LN DCs and macrophages after vaccinated with the nanovaccine.

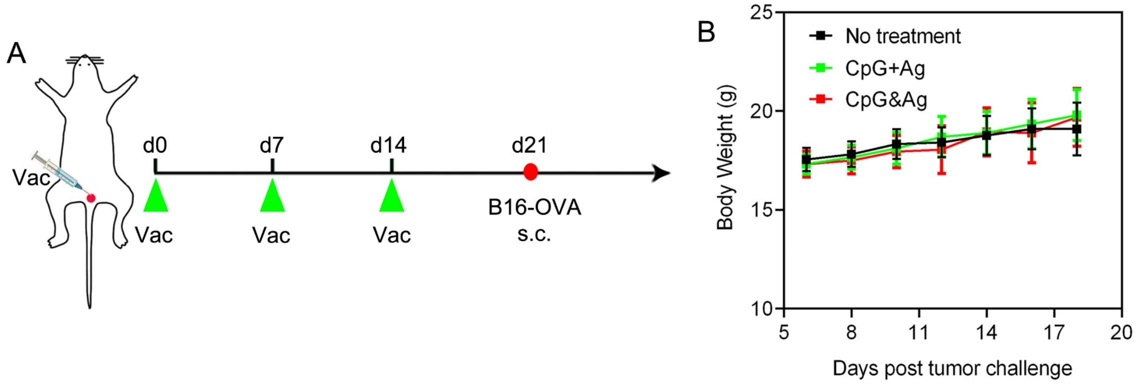

Figure S13. (A) Experimental design for B16-OVA tumor challenge study. (B) Mice body weight fluctuations monitored during the B16-OVA tumor challenge study. Data are presented as means ± s.d. (n = 5-8).

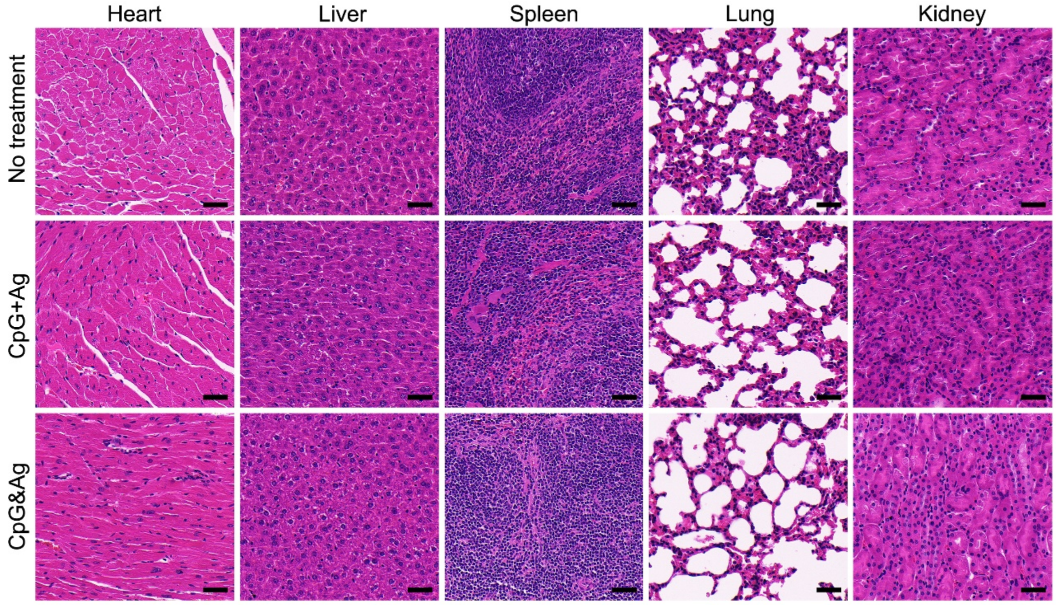

Figure S14. Representative H&E staining images of main organs harvested from the mice in B16-OVA tumor challenge studies in figure 5A. Scale bars, 40 μm.

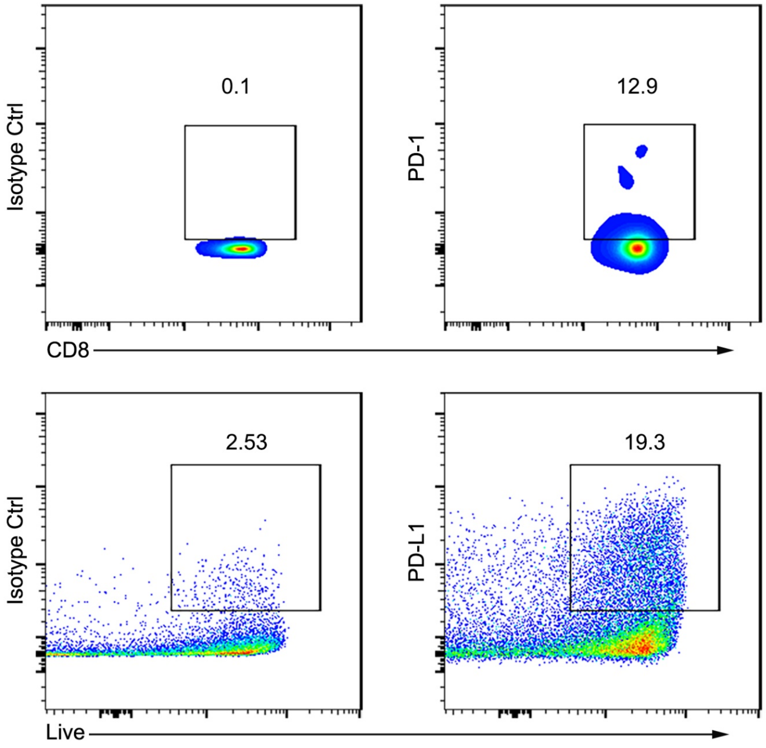

Figure S15. Flow cytometry analysis of PD-1 and PD-L1 expression levels on PBMCs and tumor cells from the B16-OVA antitumor immunotherapy experiment shown in Figure 5E.

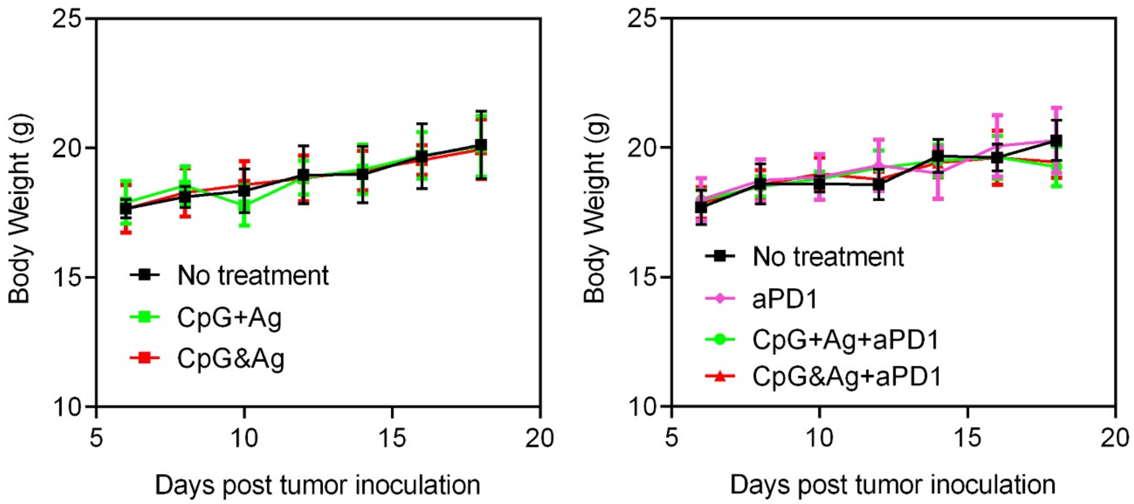

Figure S16. Mice body weight fluctuations monitored during the B16-OVA antitumor immunotherapy experiment shown in Figure 5E and 5F. Data are presented as means ± s.d. (n = 5-8).

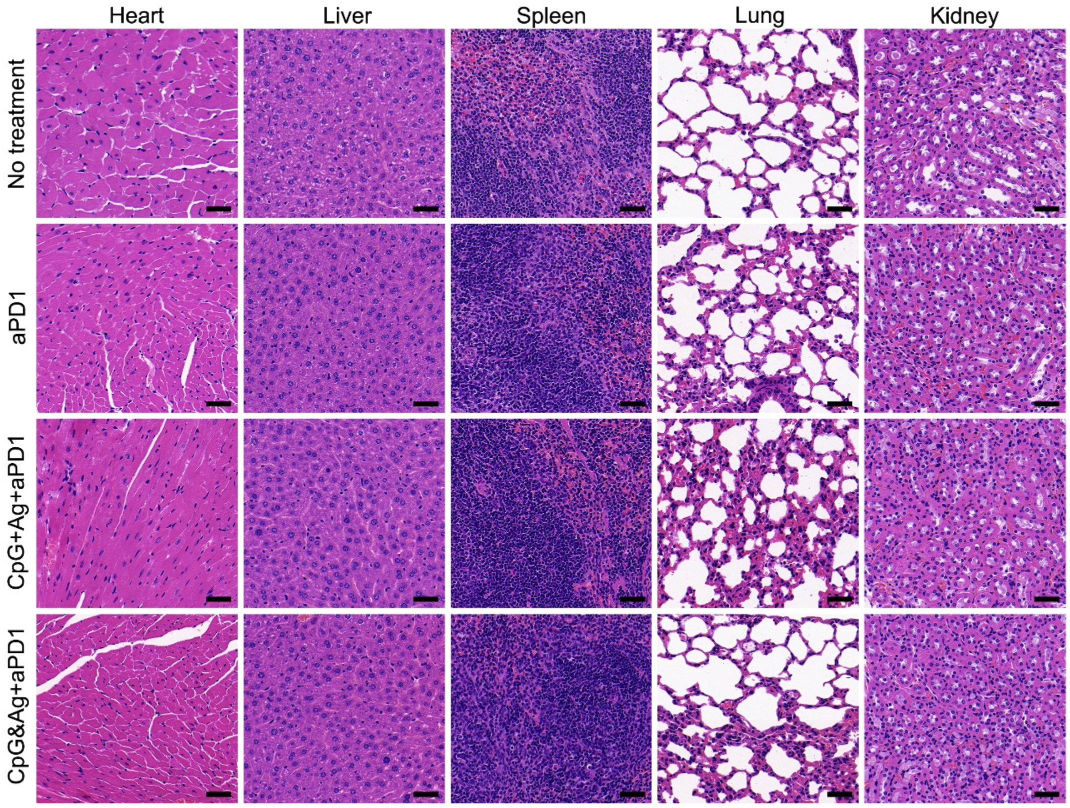

Figure S17. Representative H&E staining images of main organs harvested from the mice in B16-OVA antitumor immunotherapy study in Figure 5E and 5F. Scale bars, 40 μm.

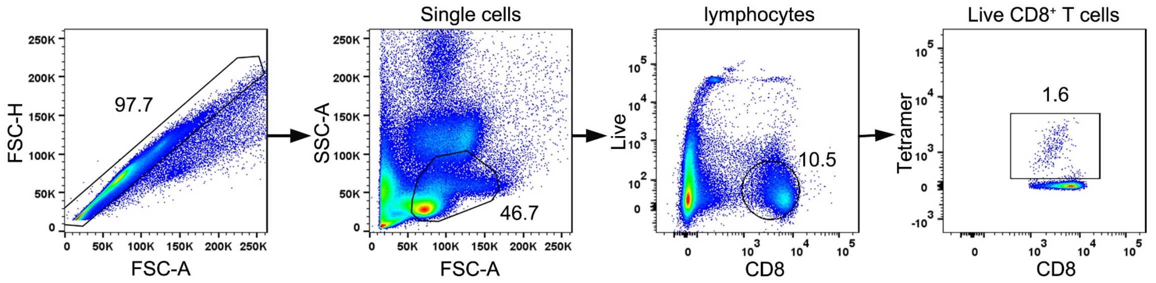

Figure S18. Gating tree exploited for flow cytometric evaluation of Adpgk-specific $CD8^+$ T cells in tetramer staining analysis in Figure 6B.

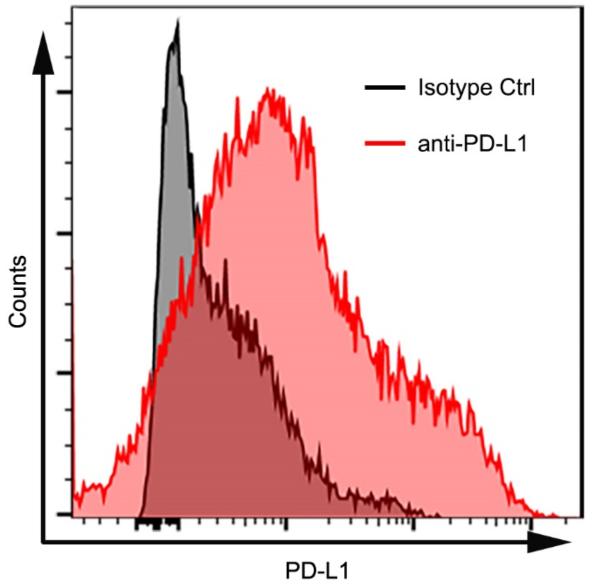

Figure S19. Flow cytometry analysis of PD-L1 expression level on tumor cells from the MC-38 antitumor immunotherapy experiment shown in Figure 6A.

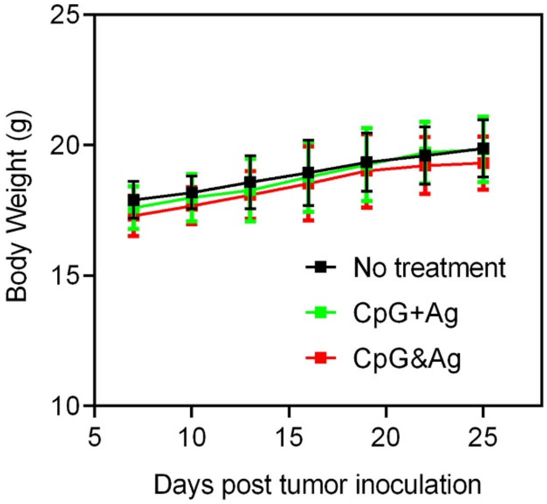

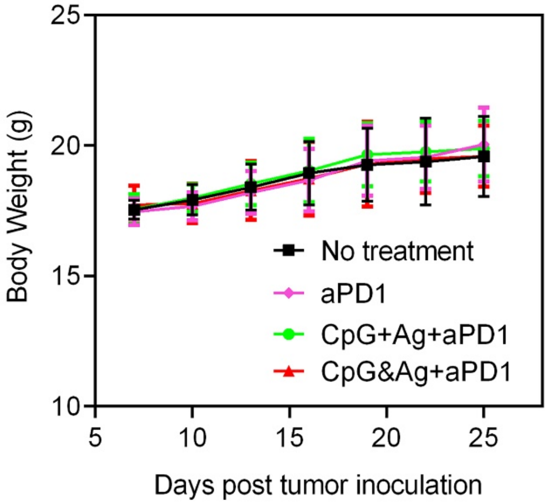

Figure S20. Mice body weight fluctuations monitored during the MC-38 antitumor immunotherapy experiment shown in Figure 6A and 6C. Data are presented as means ± s.d. (n = 5-8).
